# The VAMP-associated protein VAPB is required for cardiac and neuronal pacemaker channel function

**DOI:** 10.1096/fj.201800246R

**Published:** 2018-06-07

**Authors:** Nicole Silbernagel, Magdalena Walecki, Martin K.-H. Schäfer, Mirjam Kessler, Mehrnoush Zobeiri, Susanne Rinné, Aytug K. Kiper, Marlene A. Komadowski, Kirsty S. Vowinkel, Konstantin Wemhöner, Lisa Fortmüller, Marcus Schewe, Amalia M. Dolga, Jelena Scekic-Zahirovic, Lina A. Matschke, Carsten Culmsee, Thomas Baukrowitz, Laurent Monassier, Nina D. Ullrich, Luc Dupuis, Steffen Just, Thomas Budde, Larissa Fabritz, Niels Decher

**Affiliations:** * Institute of Physiology and Pathophysiology, Vegetative Physiology, Phillips University, Marburg, Germany;; † Institute of Anatomy and Cell Biology, Philipps University, Marburg, Germany;; ‡ Molecular Cardiology, Department of Internal Medicine II, University Hospital Ulm, Ulm, Germany;; § Institute for Physiology I, University of Münster, Munster, Germany;; ¶ Department of Cardiology II - Electrophysiology, University Hospital Münster, University of Münster, Munster, Germany;; ‖ Institute of Physiology, Christian-Albrechts University, Kiel, Germany;; # Institute of Pharmacology and Clinical Pharmacy, Phillips University, Marburg, Germany;; ** Laboratoire de Pharmacologie et Toxicologie NeuroCardiovasculaire, Faculté de Médecine, Université de Strasbourg, Strasbourg, France;; †† Institute of Physiology and Pathophysiology, University of Heidelberg, Heidelberg, Germany; ‡‡ Laboratoire de Neurobiologie et Pharmacologie Cardiovasculaire, Faculté de Médecine, Université de Strasbourg, Strasbourg, France;; §§ INSERM, Faculté de Médecine, Université de Strasbourg, Strasbourg, France;; ¶¶ Institute of Cardiovascular Sciences, University Hospital Birmingham, University of Birmingham, Birmingham, United Kingdom;; ‖‖ Department of Cardiology, University Hospital Birmingham, University of Birmingham, Birmingham, United Kingdom;; ## Division of Rhythmology, Department of Genetic Epidemiology, University Hospital Münster, University of Münster, Munster, Germany; *** Institute of Human Genetics, Department of Genetic Epidemiology, University Hospital Münster, University of Münster, Munster, Germany

**Keywords:** HCN channels, ALS, cardiac arrhythmia

## Abstract

Hyperpolarization-activated cyclic nucleotide–gated (HCN) channels encode neuronal and cardiac pacemaker currents. The composition of pacemaker channel complexes in different tissues is poorly understood, and the presence of additional HCN modulating subunits was speculated. Here we show that vesicle-associated membrane protein–associated protein B (VAPB), previously associated with a familial form of amyotrophic lateral sclerosis 8, is an essential HCN1 and HCN2 modulator. VAPB significantly increases HCN2 currents and surface expression and has a major influence on the dendritic neuronal distribution of HCN2. Severe cardiac bradycardias in VAPB-deficient zebrafish and VAPB^−/−^ mice highlight that VAPB physiologically serves to increase cardiac pacemaker currents. An altered T-wave morphology observed in the ECGs of VAPB^−/−^ mice supports the recently proposed role of HCN channels for ventricular repolarization. The critical function of VAPB in native pacemaker channel complexes will be relevant for our understanding of cardiac arrhythmias and epilepsies, and provides an unexpected link between these diseases and amyotrophic lateral sclerosis.—Silbernagel, N., Walecki, M., Schäfer, M.-K. H., Kessler, M., Zobeiri, M., Rinné, S., Kiper, A. K., Komadowski, M. A., Vowinkel, K. S., Wemhöner, K., Fortmüller, L., Schewe, M., Dolga, A. M., Scekic-Zahirovic, J., Matschke, L. A., Culmsee, C., Baukrowitz, T., Monassier, L., Ullrich, N. D., Dupuis, L., Just, S., Budde, T., Fabritz, L., Decher, N. The VAMP-associated protein VAPB is required for cardiac and neuronal pacemaker channel function.

Hyperpolarization-activated cyclic nucleotide-gated (HCN) channels play a major role in generating neuronal and cardiac automaticity (1). Upon hyperpolarization, HCN channels induce an unspecific cationic conductance, mediated by Na^+^ and K^+^ (2), described as *I*_h_ and *I*_q_ in the brain or *I*_f_ in the heart. These pacemaker currents regulate the heart rate (3), as well as rhythmic activity in neuronal circuits (4, 5) and synaptic transmission (6) but are also important for setting the resting membrane potential (7). The conserved cyclic nucleotide–binding domain (CNBD) in the C terminus of HCN channels (8) confers a cAMP sensitivity to the channels (9, 10) that can be antagonized by allosteric competition with the auxiliary HCN subunit Trip8b (11, 12). However, remaining differences in voltage dependence and pharmacology of pacemaker currents in native tissues toward the 4 known HCN channels suggest the presence of additional HCN channel–modulating proteins.

The present article describes a novel interaction partner and modulator of HCN1 and HCN2, the vesicle-associated membrane protein (VAMP)-associated protein B (VAPB or ERG30) (13). VAPs were initially described as interacting partners of VAMP, participating in neurotransmitter release (14). VAPs are, in addition to the endoplasmic reticulum, found in many intracellular compartments including, for example, the Golgi apparatus, recycling endosomes, and tight junctions, but also the plasma membrane (15, 16). Both family members, VAPA (VAP-33) (17) and VAPB, form a complex *via* their transmembrane (TM) domains and are part of the vesicle (v)-synaptosome-associated protein receptor (SNARE) complex (16). It has been proposed that VAPB is not directly participating in synaptic vesicle exocytosis but rather in the targeting of components to the synaptic terminals (18). In addition, previous studies of Kv2.1 and VAMP (19), TASK-1 and syntaxin 8 (20), or GLUT4 and VAPA (15) indicated that components of the v-SNARE or target (t)-SNARE complex (t-SNARE) might have functions in addition to regulating exocytosis, as they modulate channels or transporters that are integral to the surface/cell membrane.

## MATERIALS AND METHODS

### Split-ubiquitin yeast 2-hybrid experiments

A split-ubiquitin yeast 2-hybrid (Y2H) membrane protein system (Y2H Membrane Protein Kit; MoBiTec, Göttingen, Germany) was used for screening a NubG fused human brain cDNA library in the yeast strain NMY51 to identify potential interaction partners of HCN2. For this purpose, full-length mHCN2 was subcloned by *Sfi*I restriction sites into the bait vector pBT3-N according to the instruction manual (MoBiTec). pAl-Alg5 was used as positive control and pPR3-N as negative control. Yeast clones were selected by activation of reporter genes upon positive interaction of bait (pBT3-mHCN2) and prey (NubG-x fused cDNA library) encoding 2 auxotrophic growth markers (HIS3 and ADE2). Activation of reporter genes concedes yeast clones to grow on minimal medium lacking histidine (H), adenine (A), leucine (L), and tryptophan (W) for selection (-LWHA medium). Further selection for the strongest interactors was performed by using a β-galactosidase assay due to the property of positive colonies expressing the protein β-galactosidase. Isolated plasmid-DNA of positive yeast colonies was analyzed by sequencing. For the confirmation of interaction, a split ubiquitin Y2H direct interaction assay was performed. Therefore, full-length hVAPB was subcloned *via* *Sfi*I restriction sites into the prey vector pPR3-N and tested for an interaction with pBT3-mHCN2 or pBT3-hHCN4 by validation of colony growth on minimal medium (-LWHA).

### Expression constructs

For oocyte expression, hHCN1, mHCN2, hHCN4, and hVAPB were subcloned into pSGEM. ^NTK^HCN2 was cloned into ALMV vector and mHCN2^HA^_Ex_ into pBF1. cRNA was prepared with an mMessage mMachine T7 or SP6 Kit (Thermo Fisher Scientific, Waltham, MA, USA) after linearization. The QuikChange Site-Directed Mutagenesis Kit (Stratagene; Biocompare, South San Francisco, CA, USA) was used to introduce specific mutations. hVAPB, hVAPA, hVAMP1, and hVAMP2 were subcloned in frame into the pGEX-4T-1 vector (Amersham; GE Healthcare, Freiburg, Germany) for glutathione *S*-transferase (GST) fusion protein expression in BL21 cells. VAPB and VAPA were subcloned *via* *Eco*RI and *Sal*I, whereas VAMP1 and VAMP2 were subcloned with *Bam*HI and *Eco*RI. Constructs for mammalian cell expression were subcloned in pEGFP, pDsRed, or pcDNA3.1(+) vectors.

### GST pull-down experiments

GST fusion proteins were obtained after lysis of 1 ml BL21 cell culture. Protein expression was induced at 0.4–0.6 OD_600_ by 1 mM isopropyl β-d-thiogalactopyranoside for 3 h at 37°C. Protein extraction of a 1-ml culture was performed by using 200 µl RIPA buffer supplemented with 4 µl protease inhibitor cocktail (Sigma-Aldrich, Merck, München, Germany) and 1% lysozyme. Six freeze and thaw cycles were performed to improve the protein yield. HCN2 protein was prepared by lysis of HeLa cells transfected with 2 µg HCN2^EGFP^. HeLa cells were harvested 48 h after transfection, washed in PBS, and lysed 30 min on ice using 200 µl RIPA buffer supplemented with 10 µl protease inhibitor cocktail (Sigma-Aldrich). Insoluble material was separated by centrifugation (13,000 rpm for 15 min at 4°C). The TnT Quick Coupled Transcription/Translation System (T7; Promega, Madison, WI, USA) was used to synthesize *in vitro* translated HCN2 based on HCN2 pcDNA3.1 plasmid using T7 promotor. Pull-down experiments were performed following the MagneGST Pull-Down System (Promega) manual. Proteins were eluted from magnetic beads 2 times with 20 µl SDS sample buffer. For separation of the proteins by SDS-PAGE, pull-downs were heat denatured at 40°C for 10 min. After centrifugation at 5000 rpm for 3 min, protein lysates were separated on an 8% SDS-polyacrylamide gel. The input was diluted 1:15 and 1:10. Proteins were transferred to a nitrocellulose membrane *via* a Semi-Dry Transfer Cell (Bio-Rad, Munich, Germany) at 70 mA for 1 h. For GST pull-downs with HCN2^EGFP^, the HCN2 protein was visualized by immunoblotting with mouse α–green fluorescent protein (GFP) antibody (ab290, 1:5000; Abcam, Cambridge, United Kingdom). The binding of the primary antibody was detected by using peroxidase-conjugated goat α-rabbit IgG antibody (32460, 1:2000; Pierce, Thermo Fisher Scientific) and a chemiluminescent extended-duration substrate (Super Signal West Dura; Thermo Fisher Scientific). Untagged HCN2 protein was detected with rabbit α-HCN2 antibody (APC-030, 1:300; Alomone Labs, Jerusalem, Israel) and peroxidase-conjugated goat α-rabbit IgG antibody (32460, 1:2000; Thermo Fisher Scientific) as the secondary antibody.

### Two-electrode voltage-clamp measurements in *Xenopus* oocytes

Isolation of *Xenopus laevis* oocytes and two-electrode voltage-clamp (TEVC) recordings were performed as previously described (21). Stage IV and V oocytes were injected with 7.5 ng of HCN2 cRNA or coinjected with 1 ng of VAPB, VAPB^P56S^, VAPA, VAPC, VAMP1, and VAMP2, as well as 1 ng of the following VAPB mutations: TM^VAPB^, MSP^VAPB^, MSP-CC_0.5_^VAPB^, and MSP-CC^VAPB^. For the C-terminal deletion constructs, 3.5 ng ^NTK^HCN2, HCN2^F530*^, and HCN2^V628*^ or 15 ng of HCN2^F486*^were coexpressed with 1 ng VAPB. To determine selectivity within HCN-channel family members, 3.5 ng HCN1 or 15 ng HCN4 cRNA was coexpressed with 1 ng VAPB; 3.5 ng HCN2 was coexpressed with 0.25 ng VAPA/B complex. TEVC experiments were performed in ND96 solution, containing (mM): NaCl 96, KCl 2, CaCl_2_ 1.8, MgCl_2_ 1, HEPES 5; pH 7.4, 2 d after injection of the respective constructs. ND66 solution, containing (mM): NaCl 66, KCl 32, CaCl_2_ 1.8, MgCl_2_ 1, HEPES 5; pH 7.4, was used for the coexpression experiments of VAPB with HCN4, HCN2^F486*^, and the complex measurements of HCN2 and VAPA/B, due to small current amplitudes. Current–voltage (*I–V*) curves were recorded by 2 s voltage steps to potentials ranging from −30 to −140 mV from a holding potential of −30 mV. Tail currents were recorded by a 750 ms test pulse to −130 mV.

### Inside-out macropatch-clamp recordings from *X. laevis* oocytes

Giant patch recordings in the inside-out configuration under voltage-clamp conditions were made at room temperature (22–24°C). Pipettes were made from thick-walled borosilicate glass, had resistances of 0.3–0.5 MΩ (tip diameter of 5–15 µm), and were filled with a pipette solution containing (mM): KCl 120, HEPES 10 and CaCl_2_ 1.0; pH 7.2. Bath solutions had the following composition (mM): KCl 100, HEPES 10, K_2_EGTA 10; pH 7.2. Bath solutions were applied *via* a multibarrel pipette system to the cytoplasmic side of excised membrane patches. Currents were recorded with an EPC10 amplifier (Heka Electronik, Lambrecht, Germany), and capacitive transients were compensated with an automated circuit of the EPC10.

### Fluorescence microscopy

HeLa or HL-1 cells were grown on 35 mm glass bottom Petri dishes (WillCo, Amsterdam, The Netherlands) to a confluency of ∼50%. HeLa cells were grown in DMEM medium containing 10% fetal bovine serum (Thermo Fisher Scientific) and 1% Pen Strep (Thermo Fisher Scientific). After 24 h, HeLa cells were transfected with either 1 µg EGFP-C1-HCN2-HA_Ex_ (^EGFP^HCN2^HA^_Ex_) or cotransfected with VAPB pcDNA3.1 or TM^VAPB^ pcDNA3.1 per dish using jetPrime (Peqlab, Erlangen, Germany). Cells were maintained at 37°C for 6–48 h aerated with 5% CO_2_. For fluorescence imaging, HL-1 cells were fixed with methanol, whereas for HeLa cells, live cell imaging in an HBSS buffer (Thermo Fisher Scientific) was performed. Fixed HL-1 cells were stained with an α-VAPB antibody (sc-98992, 1:50; Santa Cruz Biotechnology, Heidelberg, Germany) after permeabilization (0.25% Triton-X-100/PBS) and blocking with 3% goat serum/PBS. For visualization, an Alexa Fluor 488 goat α-rabbit antibody (A11008, 1:200; Thermo Fisher Scientific) was used. Fluorescence microscopy was performed with an Olympus IX71 microscope equipped with a ×60 N.A. 1.3 PL APO objective or a 100× N.A. 1.4 PL APO objective (Olympus, Hamburg, Germany), standard EGFP/Texas Red filter sets, and a cooled 12-bit CCD camera (SensiCam QE; Cooke, Auburn Hills, MI, USA). During live cell imaging, cells were maintained at 37°C using an objective heater (Bioptechs, Butler, PA, USA). Digital images were processed by using Image-Pro Plus 6.0 (Media Cybernetics, Cambridge, United Kingdom).

### Analyses of channel surface expression in oocytes and HeLa cells and Western blot analysis

*X. laevis* oocytes were injected with 10 ng HCN2^HA^_Ex_ cRNA transcribed from pBF1 alone or coexpressed with 1 ng VAPB 48 h before surface expression analysis. Subsequently, a chemiluminescence assay was performed as previously described (21).

For surface expression studies in HeLa cells, cells were grown to a confluency of 90% and transfected with either 1 µg ^EGFP^HCN2^HA^_Ex_ alone or cotransfected with 1 µg VAPB pcDNA3.1 or TM^VAPB^ pcDNA3.1. Cells were fixed with 4% paraformaldehyde for 20 min on ice and blocked with 10% normal goat serum/PBS with 0.1% sodium azide for 1 h at room temperature. Immunolabeling was performed with an α-HA antibody (sc-7392, 1:100 in PBS; Santa Cruz Biotechnology) for 1 h followed by 4 times washing in PBS. After incubation with a peroxidase-coupled secondary antibody (sc-2005, α-goat, 1:5000; Santa Cruz Biotechnology), luminescence was measured in a luminometer (GloMax 20/20; Promega) by addition of chemiluminescence substrate (SuperSignal ELISA Femto Maximum Sensitivity Substrate; Thermo Fisher Scientific). Peak luminescence was determined and normalized to ^EGFP^HCN2^HA^_Ex_ mean luminescence. For determination of the whole-cell protein concentration, cells were lysed with RIPA buffer as previously described. Equal amounts of protein lysates verified by Bradford assay were separated on a 10% SDS-polyacrylamide gel with Mini-Protean Tetra Cell (Bio-Rad) at 35 mA and transferred to a nitrocellulose membrane for Western blot analysis. ^EGFP^HCN2^HA^_Ex_ was detected with an α-GFP antibody (ab290, 1:5000; Abcam).

### Preparation and transfection of primary cortical neurons

Primary cultures of cortical neurons were prepared from embryonic Sprague-Dawley rats (E16) (*Rattus norvegicus*) essentially as previously described (22). Primary cortical neurons were seeded at a density of 200,000 cells/well (24-well plates) onto polylysine-coated coverslips in Neurobasal-A medium supplemented with 2% B27, 1 µg/µl gentamicin, 2 mM GlutaMax (Thermo Fisher Scientific), and 5% fetal calf serum. After 4 h, this medium was replaced with Neurobasal medium supplemented with 1.2 mM glutamine, 2% (v/v) B27 supplement (20 ml/L), and penicillin/streptomycin (0.1 mg/ml), which was used as a culture medium. Following 3-DIV, neurons were transfected with 1 µg HA-tagged VAPB (^HA^VAPB) or ^HA^VAPB^P56S^ plasmid using 2 µl/ml Lipofectamine 2000 (Thermo Fisher Scientific) according to the manufacturer’s instructions.

Images were acquired by using a confocal laser scanning microscope (Leica SP5; Leica Microsystems, Wetzlar, Germany). Light was collected through a ×63 1.4 NA oil immersion lens with an additional ×2 optical zoom applied for dendritic regions. For green detection, cells were excited at 488 and 543 nm, and emissions were detected by using 505–530 band pass filter (green), Alexa 568 stained HCN2 or VAPB was detected by excitation at 620 nm band pass filter, and emission by using a 690 nm long pass filter (red).

### *In situ* hybridization

To generate specific *in situ* hybridization (ISH) probes, cDNA fragments of HCN2 (nt. 1404-1934, NM_008226.2), VAPA (nt. 894-1462, NM_013933.3), and VAPB (nt. 3299- 4320, NM_019806.5) were obtained by RT-PCR cloning from RNA extracts of mouse brain and subcloned into pGEM-T (Promega) for *in vitro* transcription. Specific primer sets are listed in Supplemental Table 1. Fourteen-micrometer-thick frozen brain sections were cut on a Leica cryostat, thaw-mounted on adhesive slides, and subjected to the prehybridization procedure as described. Digoxigenin (DIG)-labeled probes in anti-sense and sense orientation were generated from the templates described earlier by *in vitro* transcription using SP6 or T7 RNA polymerase in a DIG labeling mix containing 10 mM each of ATP, CTP, and GTP, 6.5 mM UTP, and 3.5 mM DIG-11-UTP (Roche, Mannheim, Germany). Riboprobes in sense orientation served as negative controls. After limited alkaline hydrolysis (0.2 M Na_2_CO_3_, pH 10.2), probes were added to the hybridization solution (3 times saline-sodium citrate [SSC], 50 mM Na_3_PO_4_, 10 mM dithiothreitol, 1 time Denhardt’s Solution, 0.25 g/L yeast tRNA, 10% dextran sulfate, and 50% formamide) to a final concentration of 0.1 to 0.5 ng/μl. To each slide, 50 µl hybridization mix was applied and incubated for 14 h at 60°C. Slides were washed in 2× SSC for 20 min, followed by 30 min RNase A treatment [20 μg RNase A and 1 U/ml RNase T1 (Roche) in 10 mM Tris-HCl, pH 8.0, 0.5 M NaCl, and 1 mM EDTA] at 37°C. The slides were washed at room temperature in decreasing salt concentrations (1, 0.5, and 0.2× SSC) for 20 min each and at 60°C in 0.2× SSC for 1 h, followed by a final rinse at room temperature in 0.2× SSC and distilled water for 10 min each.

For hybrid detection, slides were equilibrated for 30 min at room temperature in buffer A (100 mM Tris and 150 mM NaCl, pH 7.5) containing 0.05% Tween 20 (Merck, Darmstadt, Germany) and blocking reagent (Roche). Alkaline phosphatase–conjugated anti-DIG Fab fragments (11093274910; Roche) were diluted to 0.25 U/ml in blocking buffer and applied to slides for 2 h at 37°C. Excessive antibody was removed by two 10 min washes in buffer A. Slides were equilibrated to buffer B (100 mM Tris, 100 mM NaCl, and 50 mM MgCl_2_; pH 9.4) containing 0.05% Tween 20 for 10 min. The chromogen solution containing 0.2 mM 5-bromo-4-chloro-3-indolyl phosphate and 0.2 mM nitroblue tetrazolium salt (Roche) in buffer B was applied for 3 h in the dark, under periodic microscopic monitoring of color development, until reaction was stopped in water. Slides were embedded in aqueous mounting medium (Merck). Only adult (3–6 mo) in-house bred male C57BL6/J mice were used (*n* = 4). Each ISH experiment was performed 3 times, and for each brain region investigated, 9–12 sections (equals 3–4 slides) were hybridized with anti-sense probes and 6 sections (2 slides) with sense probes. For analysis and documentation, an Olympus AX70 fluorescence microscope (Olympus Optical) equipped with a Spot Digital Camera System (Diagnostics Instruments, Sterling Heights, MI, USA) was used.

### VAPA and VAPB knock-down experiments in embryonic zebrafish

Zebrafish (*Danio rerio*) of the Tübingen strain were bred and maintained at 28.5°C as described by Westerfield (23). Pictures and movies were recorded at 72 h postfertilization (hpf). Heart rate was counted at 72 hpf at room temperature. For all injection procedures for morpholino-modified antisense oligonucleotides (MO), the TE wild-type strain was used. MO (Gene Tools, Philomath, OR, USA) were directed against the translational start site (5′-TCAGGATCTGCTCCAGTTTGGACAT-3′) of zebrafish VAPA (^MO^VAPA) and the translational start site (5′-CCATCTCCCACTGCAAACGCTCGGA-3′) of zebrafish VAPB (^MO^VAPB). For rescue experiments, sense-capped cRNAs of human wild-type VAPA as well as wild-type and mutant VAPB^P56S^ were synthesized by using the mMESSAGE mMACHINE system (Ambion; Thermo Fisher Scientific). Each cRNA was, with a concentration of 75 ng/µl, coinjected with ^MO^VAPB or KCl. Calcium imaging was performed as previously described (24, 25). Wild-type and ^MO^VAPA/VAPB morphant embryos were injected with 1 nl of a 250 μM stock solution of calcium green-1 dextran (Molecular Probes, Eugene, OR, USA) at the 1-cell stage. At 72 hpf, videos were recorded with a Proxitronic (ProxiVision, Bensheim, Germany) camera at 29.97 frames/s. Relative fluorescence of the atrium and ventricle were analyzed with custom-made software (26). All zebrafish injection procedures were repeated at least 3 times (3 biologic replicates). All surviving injected embryos per group were subjected to functional analyses at 72 hpf.

### Immunostaining of VAPB in embryonic zebrafish hearts

Dissected hearts of zebrafish embryos at 72 hpf were fixed in 4% methanol-free formaldehyde in PBS. After blocking in 10% goat serum in PBS with Tween 20, hearts were incubated with a custom-made rabbit polyclonal anti-mouse VAPB antibody (1:2000), previously described (27), for 1 h at room temperature. The secondary antibodies were diluted 1:1000 in PBS with Tween 20 and incubated for 30 min at room temperature. A Zeiss Axioskop 2 plus and the AxioVision software (Zeiss, Wetzlar, Germany) were used for documentation.

### RT-PCR and ISH

RNAs from embryonic zebrafish hearts were extracted by using the RNeasy Mini Kit (Qiagen, Hilden, Germany) according to the manufacturer’s instructions. Reverse transcription was performed by using SuperScript III Reverse Transcriptase (Thermo Fisher Scientific). *Vapa*- and *vapb*-specific PCR analyses were performed according to standard protocols using SYBR-Green Master Mix (Roche) and a Roche LightCycler 480 II. Whole-mount ISH was performed by using DIG-labeled antisense RNA probes against zebrafish vapa and vapb as previously described.

### Electrocardiograms in VAPB^−/−^ mice

Noninvasive surface electrocardiogram (ECGs) from adult VAPB (+/+, +/−, −/−) mice (C57Bl/6N tac strain) gently restrained by an ECG tunnel system (EMKA Technologies, Paris, France) were recorded by using contact electrodes inserted in the tunnel floor in conscious mice. VAPB knock-out mice were backcrossed over 5 generations, and wild-type littermates served as controls for the experiments. All parameters were measured from lead I by blinded observers.

### Experiments in HL-1 cells

HL-1 cells were grown on 35 mm dishes (Nunc; Thermo Fisher Scientific) to a confluency of ∼50%. Cells in each dish were either transfected with 2 µg pEGFP vector alone, or in combination with 2 µg VAPB pcDNA3.1(+) or 2 µg pcDNA3.1(+). For knock-down experiments, HL-1 cells were transfected with either 2 µg shVAPB-GIPZ or 2 µg shcontrol-GIPZ. For Western blot analysis of HL-1 cardiomyocytes, the cells were treated with puromycin to select for transfected cells. After 24 h, HL-1 cells were measured with an EPC10 amplifier (HEKA Electronik) in the whole-cell configuration at room temperature (22°C) using previously described solutions (28). Pipettes had a tip resistance of 2.5–4.0 MΩ when filled with a solution for *I*_f_ recordings containing (mM): KCl 120, TEA-Cl 10, Na_2_GTP 0.4, Na_2_ATP 5, MgCl_2_ 2, EGTA acid 11, CaCl_2_ 5 and HEPES 5; pH 7.2. Cells were bathed in modified Tyrode solution containing (mM): NaCl 140, KCl 25, MgCl_2_ 1.2, CaCl_2_ 1.8, glucose 10, NiCl_2_ 2, BaCl_2_ 2, 4-aminopyridine 0.5 and HEPES 5; pH 7.4. Series resistances were automatically compensated by 70%. *I–V* relationships were recorded by 1.5 s voltage steps ranging from −20 to −120 mV in 20 mV increments, recorded from a holding potential of −30 mV with a sweep time interval of 10 s. Tail currents were elicited by a 300 ms step to −110 mV.

For action potential measurements, HL-1 cells were grown to 100% confluency, and action potentials were measured in the current clamp mode without electrical stimulation. Pipettes had a resistance between 2.5 and 4.0 MΩ, when filled with a solution containing (mM): KCl 60, K-glutamate 65, Na_2_GTP 0.2, K_2_ATP 3, MgCl_2_ 2, EGTA 5, and HEPES 5; pH 7.2. Whole-cell measurements were obtained at room temperature in a bath solution containing (mM): NaCl 135, KCl 5, MgCl_2_ 1, NaH_2_PO_4_ 0.33, glucose 10, Na-pyruvate 2, CaCl_2_ 1 and HEPES 10; pH 7.4.

### Slice patch-clamp experiments of neurons of the Pir and the thalamus

Animals were euthanized under isoflurane anesthesia, and brain tissue was rapidly removed from the skull. Thalamic coronal slices (250 µm) were prepared from VAPB^−/−^ and C57Bl/6N tac mice (p90-p120) on ice-cold oxygenated (O_2_) saline solution, containing (mM): sucrose 200, PIPES 20, KCl 2.5, NaH_2_PO_4_ 1.25, MgSO_4_ 10, CaCl_2_ 0.5, dextrose 10; pH 7.35. Slices were transferred to a chamber with an artificial cerebrospinal fluid containing (mM): NaCl 125, KCl 2.5, NaH_2_PO_4_ 1.25, NaHCO_3_ 24, MgSO_4_ 2, CaCl_2_ 2, dextrose 10; and kept for 30 min at the temperature of 30–32°C and further at room temperature until the recording started. pH was adjusted to 7.35 by constant aerating with carbogen (95% O_2_ and 5% CO_2_). *I*_h_ was recorded in the whole-cell mode from thalamocortical neurons of the ventrobasal thalamic complex and pyramidal neurons of the piriform cortex (Pir). Slices were transferred to a recording chamber with an external solution (bath solution) containing (mM): NaCl 120, KCl 2.5, NaH_2_PO_4_ 1.25, HEPES 30, MgSO_4_ 2, CaCl_2_ 2, dextrose 10; pH 7.25. Recordings were performed at 30 ± 1°C. Patch pipettes were pulled from borosilicate glass (GC150T-10; Clark Electromedical Instruments, Edenbridge, United Kingdom) and had resistances of 3–5 MΩ. The pipette solution contained (mM): K-gluconate 95, K_3_-citrate 20, NaCl 10, HEPES 10, MgCl_2_ 1, CaCl_2_ 0.5, BAPTA 3, MgATP 3, Na_2_GTP 0.5; pH 7.25 and 295 mOsm/kg. All recordings were made from the soma of the neurons using an EPC10 amplifier (Heka Electronik). The access resistance was in a range of 5–25 MΩ and was monitored throughout the whole experiment. Cells with access resistance >25 MΩ were discarded. Series resistance compensation of >30% was applied. Voltage-clamp experiments were controlled by the software Pulse or PatchMaster (Heka Electronik). Measurements were corrected for a liquid junction potential of 10 mV. The protocol used for assessment of *I*_h_ current is as previously described (5). Briefly, *I*_h_ current was measured by hyperpolarizing steps of −10 mV increments from a holding potential of −40 to −130 mV.

### Immunohistochemistry of HCN channels in the Pir

Twenty- to thirty-day-old C57BL/6NTac mice were transcardially perfused with 4% paraformaldehyde. Brains were removed and postfixed for 2 h in 4% paraformaldehyde and later in 30% sucrose for 48 h. Free-floating coronal sections (40 µm) were cut, and slices were collected in PBS. Sections were washed 3 times for 10 min in PBS and preincubated for 20 min in PBS containing 0.3% Triton-X100. Slices were then incubated for 2 h in 6% normal goat serum in PBS/Triton-X100 0.3%. Slices were incubated with primary antibodies: rabbit (rb)-anti-HCN1 (APC-056, 1:200), rb-anti-HCN2 (APC-030, 1:200), rb-anti-HCN4 (APC-052, 1:200) (polyclonal rabbit-antibodies; Alomone Labs), and guinea pig–anti-Map2 (188004, 1:100; Synaptic Systems, Göttingen, Germany) for 48 h at 4°C. After incubation with the primary antibody, slices were washed 3 times for 10 min in PBS and thereafter transferred to the secondary antibody solution (Alexa Fluor 568 goat anti–guinea pig IgG, ab175714, 1:1000 and Alexa Fluor 488 goat anti-rb-IgG, ab150113, 1:1000) for 1 h, washed 3 times thereafter for 10 min and mounted with a mounting medium (Vectashield with DAPI; Vector Laboratories, Burlingame, CA, USA) for confocal microscopy.

### Patch-clamp recordings of *I*_Na_ and *I*_Ca_ in induced pluripotent stem cell–derived cardiomyocytes

Whole-cell patch-clamp experiments for measurement of Ca^2+^ and Na^+^ currents were performed in murine-induced pluripotent stem cell–derived cardiomyocytes (iPSC-CMs; commercially available from Axiogenesis AG, Cologne, Germany; *http://axiogenesis.com/*). Cells were seeded in 35 mm glass bottom dishes (MatTek Corp., Ashland, MA, USA) coated with laminin and fibronectin at a density of 2 × 10^4^ cells per dish. Cells were incubated at 5% CO_2_ and 37°C with CorAt media. Cells were transfected with pEGFP empty vector (200 ng) either with pcDNA3.1(+) vector (1 µg) as control or with VAPB pcDNA3.1(+) (1 µg) using Lipofectamine 3000 (Thermo Fisher Scientific) following manual instructions. Currents were measured 48–72 h after transfection with glass electrodes (1.5–5 MΩ) filled with intracellular solution containing (mM): NaCl 8, CsAsp 120, TEA-Cl 20, MgCl_2_ 5.9, HEPES 20 and K_2_ATP 5, pH 7.2. An EPC-10 amplifier (HEKA Electronik) and the Fitmaster program (Heka Electronik) were used for data acquisition. For measurements of the Ca^2+^ currents (*I*_Ca_), cells were kept in normal tyrode solution (mM: NaCl 140, KCl 5.4, MgCl_2_ 1.1, HEPES 5, glucose 10, CaCl_2_ 1.8, pH 7.4) supplemented with CsCl (5 mM). Whole-cell currents were recorded from a holding potential of −80 mV, using a 500 ms voltage ramp from −80 to −40 mV to inactivate sodium currents before *I–V* measurements. Starting from a test potential of −40 mV, cells were clamped to different voltages in 400 ms steps ranging from −50 to 40 mV in 10 mV increments. Sodium currents (*I*_Na_) were elicited in a bath solution containing (mM): NaCl 20, NMDG 120, KCl 4, MgCl_2_ 1, CaCl_2_ 1.8, HEPES 5, glucose 10, pH 7.4, supplemented with 5 µM nifedipine. Whole-cell currents were recorded from a holding potential of −80 mV followed by 100 ms voltage steps from −80 mV to 40 mV in 5 mV increments.

### Patch-clamp recordings of Cav1.3 in HEK293 cells

HEK293 cells were cultured in 25 cm^2^ flasks at 37°C and 5% CO_2_ in DMEM (Thermo Fisher Scientific) supplemented with 10% FCS and 1% penicillin/streptomycin solution (Thermo Fisher Scientific). At a confluency of 60–70%, cells were transiently transfected with cDNA encoding Cav1.3 α_1_-subunits (human, 6 µg/flask) together with auxiliary β_2b_ (human, 3 µg/flask) and α_2_δ_1_-subunits (human, 3 µg/flask), EGFP (0.5 µg/flask), and empty vector (pcDNA3.1(+), 3 µg/flask) or VAPB (human, 3 µg/flask) using JetPrime (Peqlab). Cells were subsequently kept at 30°C and 5% CO_2_. Forty-eight hours after transfection, cells were detached by using 0.05% trypsin and transferred to 35-mm Petri dishes for electrophysiological recordings. Whole-cell patch-clamp recordings were performed at room temperature with an EPC10 amplifier (Heka Electronik) using electrodes pulled from borosilicate capillaries with a resistance of 2–5 MΩ. Series resistance was compensated by 50%. The extracellular solution contained 110 mM NaCl, 20 mM CsCl, 10 mM BaCl_2_, 10 mM HEPES, 1 mM MgCl_2_, and 10 mM glucose (pH 7.4). The pipette internal solution contained 130 mM KCl, 10 mM NaCl, 0.5 mM MgCl_2_, 1 mM EGTA, and 5 mM HEPES (pH 7.3). *I–V* relationships were obtained by applying a 300 ms long square pulse protocol to various test potentials starting from a holding potential of −80 mV.

### Cell culture

HEK293 and HeLa cells were obtained from Sigma-Aldrich and mouse iPSC-CMs from Axiogenesis, which were not authenticated again after commercial purchase. HL-1 cells were previously obtained by Prof. W. Claycomb (Louisiana State University Health Sciences Center, New Orleans, LA, USA) and authenticated by their electrophysiological properties (21). All cells were probed for mycoplasma contamination.

### Quantification and statistical analysis

Statistics were performed as previously described (29). Briefly, every dataset for wild-type and each mutant and for every current/kinetical feature analyzed were tested with a Shapiro-Wilk test for normality. Equality of variances was tested by using either a parametric or nonparametric Levene’s test. In case of similar variances, significance was probed by using either a paired or unpaired Student’s *t* test; for non-normally distributed data, a nonparametric Mann-Whitney *U* test and a Wilcoxon signed-rank test for paired analyses was used, respectively. If the variances of the groups were different, significance was probed by using Welch’s *t* test; for non-normally distributed data, Mood’s median test was used. Experiments were nonrandomized and nonblinded, and no prespecified sample size was estimated.

For inclusion/exclusion criteria, only dead embryonic zebrafish were excluded from the statistical analyses. For experiments using VAPB^−/−^ mice, estimated differences were based on previous data and calculations of sample size assuming 1 – β = 0.80 and α = 0.05. To detect changes in ECG recordings, at least 8 animals per genotype were used to ensure adequate power to detect 20–30% change. All alive and healthy-looking littermate animals were examined. ECG recordings of all examined mice was high enough in quality for analyzing heart rate, PQ interval, QT interval, and duration of QRS complex following pre-established criteria. T-wave morphology analysis of 3 mice had to be excluded because signals were divergent from pre-established criteria. The order of taking ECG recordings was randomized to consecutive order following cage numbers, which were marked by the animal facility. The investigators performing and analyzing the ECGs were blinded to the genotype.

### Ethical approval

The investigation conforms to the *Guide for the Care and Use of Laboratory Animals* (National Institutes of Health, Bethesda, MD, USA). All experimental procedures were performed in accordance with the principles approved by local authorities (Landesamt für Natur, Umwelt und Verbraucherschutz Nordrhein-Westfalen, Germany; 84-02.04.2015.A574, 84-02.05.50.15.026 and 887-50 10 37 09.125) or the United Kingdom Home Office (30/2967). Efforts were made to minimize the number of animals and the degree of discomfort to animals used in this study.

## RESULTS

### VAPB selectively increases HCN1 and HCN2 pacemaker currents

**Figure 1:**
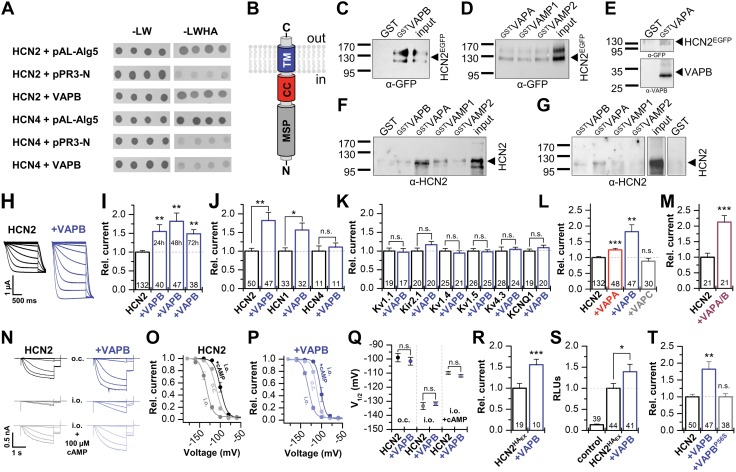
VAPB selectively increases HCN1 and HCN2 currents. *A*) Y2H direct interaction assay. Transformation control (-LW), leucine, and tryptophan dropout. Interaction read-out (-LWHA), additional dropout of histidine and adenine. pAL-Alg5, positive control. pPR3-N, negative control. *B*) Topology of VAPB. *C*) ^GST^VAPB pull-down of HCN2^EGFP^ using transfected HeLa cells. *D*) ^GST^VAPA, ^GST^VAMP1, or ^GST^VAMP2 pull-down of HCN2^EGFP^ using transfected HeLa cells. *E*) ^GST^VAPA pull-down of HCN2 and endogenous VAPB, using HCN2^EGFP^ transfected HeLa cells. *F*) Pull-down of *in vitro* translated HCN2 (untagged). *G*) Pull-down of HCN2 from rat brain lysates. *H*, *I*) Representative currents (*H*) of HCN2 expressed in oocytes alone or with VAPB and the relative current amplitudes (*I*) analyzed over 3 d. *J*) Relative currents of HCN1, HCN2, and HCN4 alone or coexpressed with VAPB. *K*, *L*) Relative currents of different potassium channels (*K*) coexpressed with VAPB and of HCN2 (*L*) coexpressed with VAPA, VAPB, or VAPC. *M*) Relative currents of HCN2 coexpressed with a mixture of VAPA/B (1:1). *N*) Representative macropatch recordings in different configurations: on cell (o.c.), inside-out after patch excision (i.o.), and after application of 100 µM cAMP (i.o.+100 µM cAMP). *O*, *P*) Activation curves for HCN2 alone (*n* = 6) (*O*), recorded as in *N*, or after coexpression with VAPB (*n* = 8) (*P*). *Q*) *V*_1/2_ values for HCN2 expressed alone or with VAPB in different patch modes. *R*) Relative currents of HCN2^HA^_Ex_ alone or with VAPB. *S*) Relative surface expression of HCN2^HA^_Ex_ expressed alone or with VAPB, analyzed as relative light units (RLUs). *T*) Relative currents of HCN2 expressed alone or with VAPB or VAPB^P56S^. All data are presented as means ± sem. The number of cells (*n*) is indicated in the bar graphs. N.s., not significant. *P < 0.05, **P < 0.01, ***P < 0.001 [unpaired Student’s *t* test (*H*, *K*, *L*, *O–S*) or Mann-Whitney *U* test (*I*, *J, M, N, T*)].

Using a split-ubiquitin Y2H (30) screen with the full-length HCN2 channel as a bait to search for plasma membrane–bound channel modulators, VAPB was identified from a human adult brain cDNA library as a potential HCN2 interacting partner. From 458 clones that grew under dropout conditions, 35 clones contained VAPB. In contrast, VAPA, the closest relative of VAPB, was not fished from the human brain cDNA library. Y2H direct-interaction assays confirmed an interaction of VAPB with HCN2 but not HCN4 (**Fig. 1*A***). VAP proteins are anchored with a C-terminal TM domain in the cell membrane, contain a conserved N-terminal major sperm region (MSP) and an amphipathic coiled-coil domain (CC), which is a common motif in t-SNAREs (31) (Fig. 1*B*). The VAPB fragments identified through the Y2H screen include the TM domain and parts of the CC domains but not the MSP domain (Supplemental Fig. 1). GST pull-down experiments confirmed an interaction of HCN2 with VAPB (Fig. 1*C*) but also the close family member VAPA and the VAP-interacting partners VAMP1 and VAMP2 (Fig. 1*D*). Using HCN2 transfected HeLa cells, ^GST^VAPA pulled down HCN2 together with endogenously expressed VAPB, suggesting that HCN2 channels might be in a complex with VAPA and VAPB (Fig. 1*E*). VAPB and VAPA both directly interact with *in vitro* translated HCN2, although the physical interaction with VAPA seems to be more efficient (Fig. 1*F*). Pull-down experiments with *in vitro* translated HCN2 also confirmed a direct interaction with VAMPs. Most importantly, both ^GST^VAPA and ^GST^VAPB pulled down HCN2 from rat brain protein lysates (Fig. 1*G*).

The coexpression of HCN2 with low amounts of VAPB in *Xenopus* oocytes strongly increased current amplitudes (Fig. 1*H, I*). HCN1 current amplitudes were also increased (Fig. 1*J*), whereas HCN4 was not modulated, consistent with our interaction analyses (Fig. 1*A, J*). None of the other tested potassium channels were influenced by VAPB, revealing a selectivity for HCN1 and HCN2 channels (Fig. 1*K*). To further support this observation, we performed patch-clamp experiments of mouse iPSC-CMs transfected with VAPB to probe for effects of VAPB on cardiac sodium and calcium channels. VAPB transfection did not alter inward sodium (*I*_Na_) or calcium (*I*_Ca_) currents (Supplemental Fig. 2*A–F* and Materials and Methods). In addition, because these iPSC-CMs reflect ventricular cardiomyocytes rather than sino-atrial pacemaker cells, we also excluded that VAPB modulates Cav1.3, a channel highly relevant for sino-atrial pacemaking (Supplemental Fig. 2*G–I*). The VAP family contains an alternative spliced isoform, VAPC, lacking the CC and the TM domains (17, 32); whereas VAPA also mildly increases the HCN2 current amplitudes, VAPC has no modulatory effect (Fig. 1*L* and Supplemental Fig. 3). Strikingly, coexpressing HCN2 together with VAPA and VAPB led to a similar and slightly more pronounced current increase (Fig. 1*M*), compared with VAPB coexpressed alone. Although the current increase was not significantly larger, the effect was more robust (HCN2 + VAPB, *P* < 0.01, *vs.* HCN2 + VAPA/VAPB, *P* < 0.001).

VAPB did not alter the voltage dependence of HCN2 activation (Supplemental Fig. 4*A*) or the activation kinetics (Supplemental Fig. 4*B*); a similar lack of effects was observed for VAPA and VAPC (Supplemental Fig. 3). Because the effects of VAPB on the voltage-dependence of activation might be missed due to the high cAMP levels intrinsic to oocytes, we performed inside-out macropatch-clamp recordings from oocytes to directly control intracellular cAMP concentrations. However, also in inside-out patches and in absence of intracellular cAMP, VAPB did not influence the voltage dependence of activation (Fig. 1*N–Q*). In addition, application of 100 µM cAMP maximally shifted the voltage dependence of activation in a similar manner, as for HCN2 alone, indicating that there is no effect of VAPB on the cAMP modulation of the channels.

We next investigated the mechanism for the increased HCN2 current amplitudes. The current amplitudes of HCN2, now harboring an extracellular HA-epitope (HCN2^HA^_Ex_), was increased, similar as described for the untagged protein (Fig. 1*R*). The surface expression in the same oocytes, probed with a chemiluminescence-assay (ELISA) exclusively detecting channels at the plasma membrane, revealed that the increased current amplitudes are mainly caused by an increased surface expression of the channels (Fig. 1*R, S*).

The previously described VAPB^P56S^ mutation located in the MSP domain causes amyotrophic lateral sclerosis (ALS) 8 and late-onset spinal muscular atrophy (33). Coexpression of VAPB^P56S^ with HCN2 revealed that the ALS-causing mutation lost its ability to increase the HCN2 current amplitudes (Fig. 1*T*).

### Domains mediating the interaction of VAPB with HCN2

**Figure 2:**
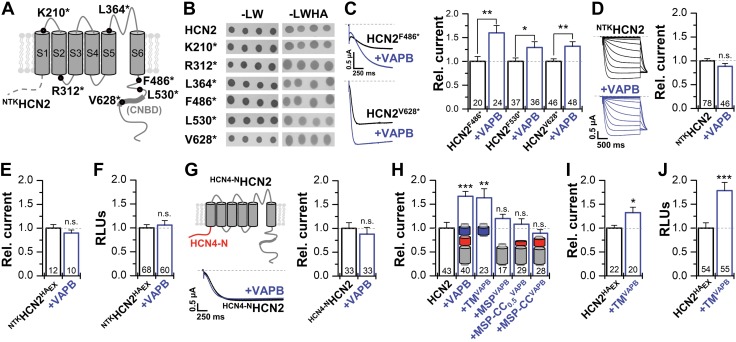
Domains mediating the interaction of VAPB with HCN2. *A*) Schematic illustration of a HCN subunit. The CNBD and some of the truncation constructs studied are indicated. *B*) All truncation constructs exhibited a positive interaction, evident from growth on -LWHA dropout medium. *C*) Representative current traces and the relative currents for different C-terminal deletions expressed alone or with VAPB. *D*) Representative current traces and the relative current amplitudes for the N-terminal truncated ^NTK^HCN2 expressed alone or with VAPB. *E*) Relative current amplitudes of ^NTK^HCN2^HA^_Ex_ (extracellular HA-tag) expressed alone or with VAPB. *F*) Relative surface expression of ^NTK^HCN2^HA^_Ex_ expressed alone or with VAPB analyzed as relative light units (RLUs). *G*) Schematic illustration, representative traces, and currents of a HCN2 channel chimera with the N terminus of HCN4 (^HCN4-N^HCN2) expressed alone or with VAPB. *H*) Relative currents of HCN2 expressed alone or coexpressed with VAPB (1.7 ± 0.1), TM^VAPB^ (1.6 ± 0.2), the MSP domain (MSP^VAPB^), the MSP with half of the CC domain (MSP-CC_0.5_^VAPB^), or with the complete CC domain (MSP-CC^VAPB^). *I*, *J*) Relative current amplitudes of HCN2^HA^_Ex_ expressed alone or with TM^VAPB^ (1.3 ± 0.1) (*I*) and the respective changes in the relative surface membrane expression analyzed as RLUs, using a single cell chemiluminescence assay (TM^VAPB^ 1.8 ± 0.2) (*J*). All data are presented as means ± sem. The number of experiments (*n*) is indicated in the respective bar graphs. N.s., not significant. *P < 0.05, **P < 0.01, ***P < 0.001 [unpaired Student’s *t* test (*E*), Mann-Whitney *U* test (*C*, *D*, *F*–*H*, *J*), or Welch’s *t* test (*I*)].

A split-ubiquitin Y2H direct-interaction assay introducing different premature stop codons in HCN2 revealed that the CNBD and the entire C terminus are dispensable for the interaction with VAPB and that the N terminus of HCN2 together with the S1 segment are sufficient for the interaction (**Fig. 2*A, B*** and Supplemental Fig. 5). These data are supported by functional studies (Fig. 2*C*) in which VAPB still increased the current amplitudes of HCN2 channels that lack the entire C terminus (HCN2^F486*^). In contrast, current amplitudes of HCN2 channels with an N-terminal deletion (^NTK^HCN2) (34) were no longer modulated by VAPB (Fig. 2*D*). Consistently, the ^NTK^HCN2^HA^_Ex_ current amplitude (Fig. 2*E*) and surface expression (Fig. 2*F*) were not altered by VAPB. Similar effects were obtained replacing the N terminus of HCN2 by that of HCN4 (^HCN4-N^HCN2), which also resulted in a loss of VAPB modulation (Fig. 2*G*).

We next probed which parts of VAPB mediate the functional effects on HCN2. Surprisingly, the TM segment of VAPB alone (TM^VAPB^) is sufficient to increase HCN2 currents (Fig. 2*H*), whereas the MSP domain alone or in combination with the CC domain (MSP+CC^VAPB^) is ineffective. These data are consistent with the lack of HCN2 modulation by VAPC, which lacks the TM domain (17). Confirming the role of the TM segment for channel modulation, the HCN2^HA^_Ex_ current amplitudes (Fig. 2*I*) and surface expression (Fig. 2*J*) were also increased by TM^VAPB^.

### VAPB determines surface expression and dendritic localization of HCN2

**Figure 3:**
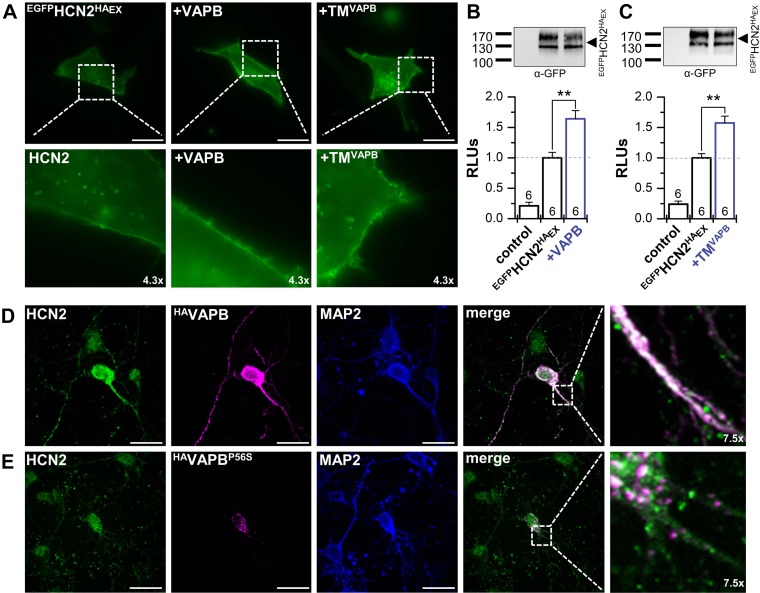
VAPB determines surface expression and dendritic localization of HCN2. *A*) Live cell imaging of HeLa cells transfected with an N-terminally EGFP-tagged HCN2 carrying an extracellular HA-epitope (^EGFP^HCN2^HA^_Ex_) alone or cotransfected with VAPB or the TM segment of VAPB (TM^VAPB^). *B*) Chemiluminescence assays of fixed non-permeabilized HeLa cells, analyzing the surface expression as relative light units (RLUs) for ^EGFP^HCN2^HA^_Ex_ alone and after cotransfection with VAPB (1.6 ± 0.1). Upper inset illustrates a representative control Western blot showing an unaltered HCN2 protein expression. *C*) Chemiluminescence surface expression assay as in *B*, but using TM^VAPB^ (1.6 ± 0.1). *D*) Immunocytochemistry of ^HA^VAPB transfected cortical neurons. Endogenous HCN2 (green) is colocalizing (white) with ^HA^VAPB (magenta) in the soma and dendrites. Anti–MAP2-staining illustrating an intact neuronal network and dendrites (blue). *E*) Immunocytochemistry experiment as in *D*, but transfecting the ALS8 mutation ^HA^VAPB^P56S^ (magenta), leading to an aggregation of VAPB^P56S^ in the soma of the neurons. Also, HCN2 fluorescence (green) was focused in the soma and dendritic localization was lost, despite an intact neuronal network (α-MAP2, blue). Scale bars, 20 µm (*A*, *D*, *E*). All data are presented as means ± sem. The number of experiments (*n*) is indicated in the respective bar graphs. **P < 0.01 (unpaired Student’s *t* test).

Cotransfection of N-terminally tagged HCN2^HA^_Ex_ (^EGFP^HCN2^HA^_Ex_) with VAPB or TM^VAPB^ resulted in increased fluorescence at the plasma membrane (**Fig. 3*A***). Untagged VAPB was used for these recordings because fluorescent protein tags reportedly cause a dimerization of VAPB (35, 36). The increased surface expression was quantified detecting HCN2 channels at the cell membrane by using nonpermeabilized, fixed cells and an ELISA assay, recognizing the extracellular HA-epitope of the ^EGFP^HCN2^HA^_Ex_ construct. Consistent with the data from the oocyte expression system (also Fig. 2*F, J* and Supplemental Fig. 1), an increased surface expression of HCN2 was observed in the presence of VAPB or TM^VAPB^ (Fig. 3*B, C*). We then probed whether the effects of VAPB on HCN2 surface expression are caused by alterations in channel endocytosis. We therefore used either dynasore or the C terminus of the adaptor protein 180 (AP180C), a construct that blocks clathrin-mediated endocytosis (20, 37). Here, despite inhibiting endocytosis by dynasore or AP180C, VAPB increased the HCN2 surface expression with a similar efficiency (Supplemental Fig. 6*A–C*). Taken together, these data support the theory that enhanced surface expression of HCN2 is most likely caused by a more efficient forward transport of the channel to the plasma membrane. Because HCN2 currents and surface expression are also increased by a minimal VAPB protein (TM^VAPB^), with a single TM domain (Figs. 2*H–J* and 3*A, C*), the most straightforward explanation is that VAPB may be part of the native HCN channel complex which is more efficiently transported to the membrane.

We next analyzed the endogenous HCN2 expression in primary cortical neurons after transfection with HA-tagged VAPB (^HA^VAPB) (Fig. 3*D*). Here, HCN2 and transfected ^HA^VAPB strongly colocalized in the dendrites and the soma of the neurons (merge to white). In contrast, the transfected ALS8 mutation ^HA^VAPB^P56S^ aggregates in the soma, consistent with previous studies (35) (Fig. 3*E*). Most importantly, HCN2 fluorescence is now primarily located in the soma, and a loss of distribution into the dendrites can be observed, although the dendrites of the neurons are still intact, as visible from the MAP2 staining of ^HA^VAPB^P56S^ transfected neurons.

### Codistribution of VAPs with HCN2 and contribution to thalamic*I*_h_

**Figure 4:**
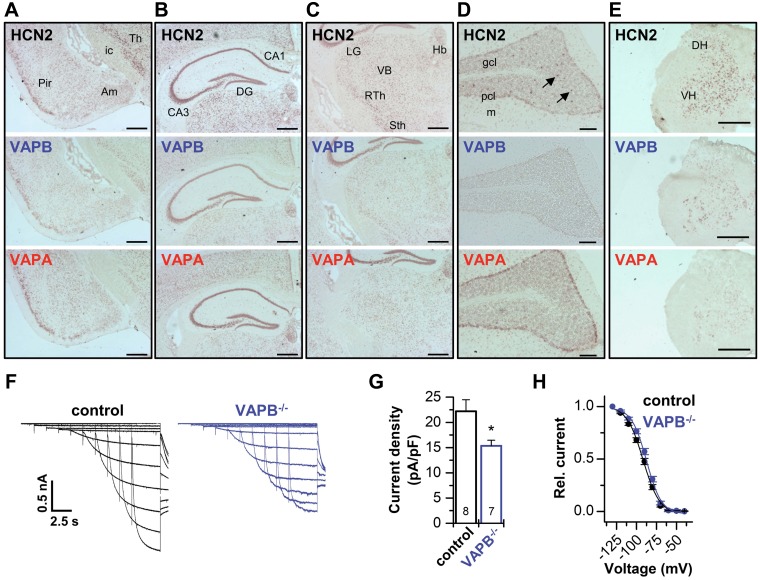
Codistribution of VAPs with HCN2 and contribution to thalamic *I*_h_. *A*–*E*), Distribution of HCN2, VAPB, and VAPA mRNA in mouse brain and spinal cord. ISH analysis of HCN2, VAPB, and VAPA using DIG-labeled riboprobes, revealing mRNA expression of VAPB in cortical areas (*A*), hippocampus (*B*), thalamus (*C*), cerebellum (*D*) (arrows point to interneurons in the granular layer), and spinal cord (*E*). Note the overlapping distribution of VAPB with HCN2 and VAPA mRNA. Am, amygdala; CA, cornu ammonis; DG, dentate gyrus; DH, dorsal horn; gcl, granule cell layer; Hb, habenulae; ic, internal capsule; LG, lateral geniculate ncl.; m, molecular cell layer; pcl, Purkinje cell layer; RTh, reticular thalamic ncl.; Sth, subthalamic ncl.; VB, ventrobasal thalamus; Th, thalamus; VH, ventral horn. *F*) Representative current traces elicited in slice patch-clamp experiments of the ventrobasal thalamus (VB) of wild-type animals (control) and VAPB^−/−^ mice. *G*) The *I*_h_ current was significantly reduced in VAPB^−/−^ mice (15.4 ± 1.1 pA/pF) compared with control animals (22.2 ± 2.3 pA/pF). *H*) Average activation curves of the VB *I*_h_ current for control and VAPB^−/−^ mice. *V*_1/2_ of activation for control (−91.6 ± 1.3 mV, *n* = 8) and VAPB^−/−^ (−87.5 ± 1.2 mV, *n* = 7). Scale bars: 500 µm (*A–C*, *E*), 100 µm (*D*). All data are presented as means ± sem. The number of experiments (*n*) is indicated in the respective bar graphs. *P < 0.05 (unpaired Student’s *t* test).

We next investigated the distribution of VAPB, VAPA, and HCN2 mRNAs in mouse brain and spinal cord by using ISH experiments (**Fig. 4*A–E***). In general, VAPB hybridization signals were weaker than those for VAPA. Highest VAPB mRNA levels were detected in the Pir (Fig. 4*A*), the CA3 region of the hippocampus (Fig. 4*B*), the hypothalamic arcuate and ventromedial nucleus, and in medial habenulae (Fig. 4*B, C*). An overlapping distribution of VAPB and HCN2 was noted in many brain regions, particularly evident in the reticular thalamic nucleus and globus pallidus (Fig. 4*C*). Hybridization signals for all 3 transcripts were observed in the subthalamic nucleus, the ventrobasal thalamic nucleus, the habenulae, and also the spinal cord (Fig. 4*E*).

Although the distribution of VAPB and HCN2 mRNA expression was very similar in all regions examined (Fig. 4*A–E*), the VAPA mRNA expression pattern was only partially overlapping in some brain regions. For example, Purkinje cells of the cerebellar cortex express relatively high mRNA levels for VAPA but only very low levels for HCN2 and VAPB (Fig. 4*D*). Most importantly, the observed coexpression of VAPB with HCN2 in the aforementioned neurons is in perfect agreement with the functional interaction we describe.

Consistent with a thalamic coexpression of HCN2 and VAPB and the role of VAPB to increase macroscopic HCN2 and HCN1 currents, patch-clamp experiments in neurons of the ventrobasal thalamus of VAPB^−/−^ mice (27) revealed significantly reduced *I*_h_ current amplitudes (Fig. 4*F, G*). Although *I*_h_ current amplitudes were reduced, no major effects on the voltage dependence of activation were observed (Fig. 4*H*). In addition, we performed patch-clamp experiments of neurons of the Pir that have, in contrast to the ventrobasal thalamus, a pronounced expression of HCN1 and reduced HCN4 levels (Supplemental Fig. 7*A–C* and Materials and Methods). We found only a minor reduction of *I*_h_ current amplitudes, and the voltage dependence was not significantly altered (Supplemental Fig. 7*D–G*). However, the speed of activation was slowed (Supplemental Fig. 7*H*), which is consistent with a reduced relative contribution of HCN1 channel to native *I*_h_ currents in the Pir. The fact that kinetics change while the overall *I*_h_ amplitudes largely remain unaltered indicate that there might be up-regulation of other channels or subunits after VAPB knock-out.

### Bradycardia in knock-down zebrafish embryos and VAPB^−/−^ mice

**Figure 5:**
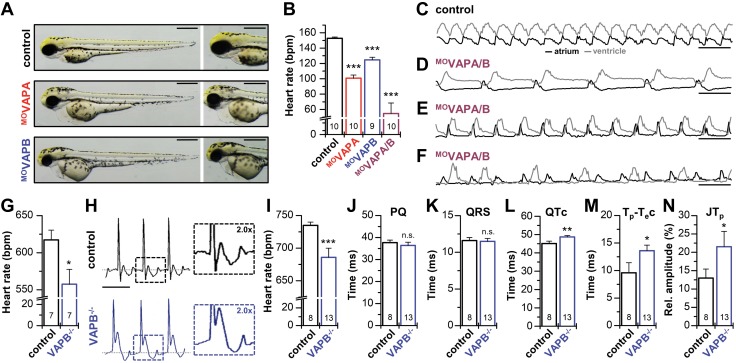
Bradycardia in knock-down zebrafish embryos and VAPB^−/−^ mice. *A*) Zebrafish embryos at 72 hpf. Control-injected and MO-injected embryos against VAPA (^MO^VAPA) or VAPB (^MO^VAPB) exhibit no significant abnormalities (left), particularly no structural heart defects (right). Note a light cardiac edema in ^MO^VAPB and a strong edema in ^MO^VAPA. *B*) Heart rate in beats per minute (bpm) of control and ^MO^VAPA, ^MO^VAPB, or ^MO^VAPA/B injected zebrafish embryos at 72 hpf. *C*) Representative examples of calcium transients (relative fluorescence intensity) in the cardiac atrium (black) and cardiac ventricle (gray) of control-injected zebrafish at 72 hpf, displaying a regular atrio-ventricular propagation of excitation from atrium to ventricle in a 1:1-ratio. *D*, *E*), Representative calcium transients recorded in ^MO^VAPA/B double knock-down morphants, illustrating strongly reduced heart rates with variable frequency. *F*) Representative example of atrial and ventricular calcium measurements from a ^MO^VAPA/B morphant with a 2:1 atrio-ventricular block, in which only every second atrial excitation leads to a ventricular excitation. Data were obtained from 3 independent batches of injections (*A*–*F*). *G*) Heart rate in beats per minute (bpm) of VAPB^−/−^ mice compared with wild-type littermates (control), analyzed by using tail-cuff measurements. *H*) Representative surface ECG recordings of VAPB^−/−^ mice and their wild-type littermates (control). ECGs of VAPB^−/−^ mice show bradycardia and an increased T-wave amplitude. *I*–*N*) Analyses of the ECG parameters of VAPB^−/−^ mice. *I*) Heart rate in beats per minute (bpm). *J*) PQ interval (PQ) duration. *K*) QRS complex (QRS) duration. *L*) Frequency corrected QT interval (QTc). *M*) Frequency-corrected T_peak_ to T_end_ duration (T_p_-T_e_c). *N*) T-wave amplitude (JT_p_). Scale bars: 500 µm (*A*); 500 ms (*C*–*F*), and 100 ms (*H*). All data are presented as means ± sem. The number of animals (*n*) is indicated in the respective bar graphs. N.s., not significant. *P < 0.05, **P < 0.01, ***P < 0.001 [Student’s *t* test (*G*, *H*, *J–N*) or Welch’s *t* test (*B*, *I*)].

ISH and RT-PCR experiments using zebrafish hearts at 72 hpf revealed a cardiac expression of both VAPA and VAPB in the embryonic heart (not illustrated). Using whole-heart immunostainings, VAPB expression was evident throughout the atrium and ventricle of the embryonic zebrafish heart (Supplemental Fig. 8*A–B*). Subsequently, to elucidate the functional role of VAPs for cardiac pacemaking *in vivo*, we inactivated either VAPB or VAPA using MO, targeting the translation initiation site of zebrafish VAPA (^MO^VAPA) or VAPB (^MO^VAPB) (**Fig. 5*A–F***). Knock-down of either VAPA or VAPB did not cause structural heart defects (data not shown). For ^MO^VAPB, a mild and for ^MO^VAPA a pronounced pericardial edema was observed (Fig. 5*A*). Moreover, a bradycardia was evident in both types of morphant zebrafish embryos (Fig. 5*B*), with 101 ± 12 beats/min for VAPA morphants and 125 ± 10 beats/min for ^MO^VAPB-injected embryos, compared with control-injected embryos with 153 ± 5 beats/min. As proof for the specificity of the knock-down, the heart rate of ^MO^VAPB-injected embryos could be rescued by the concomitant injection of either VAPA or VAPB cRNA (Supplemental Fig. 8*C*, *D*). It is noteworthy that the injection of cRNA for the human VAPB^P56S^ mutant into zebrafish embryos caused no significant reduction in the heart rate (Supplemental Fig. 8*E*), indicating that the construct does not act in a dominant-negative manner on zebrafish *I*_f_ current components. Although heart rate was significantly reduced in VAPA and VAPB morphants, both cardiac chambers, atrium and ventricle, contracted regularly and sequentially as in wild-types, whereby each atrial contraction was followed by a ventricular contraction, indicating unaffected atrio-ventricular conduction.

Bradycardia was even more severe when VAPA and VAPB were simultaneously inactivated (^MO^VAPA/B) and VAPA/VAPB morphants had severely reduced heart rates (55 ± 41 beats/min). In contrast to the VAPA or VAPB morphants, the double knock-down of VAPA and VAPB led to a broad heart rate variability (Fig. 5*D, E*) and, in 20% of all morphant embryos, to severe heart rhythm disturbances (*n* = 20) (Fig. 5*F*). To evaluate if abnormal cardiac excitation propagation or excitation-contraction-coupling account for the bradycardia and heart rhythm disturbance, we monitored free cytosolic myocardial calcium and cardiac contraction simultaneously. In 80% of VAPA/VAPB morphants (*n* = 20), cardiac excitation starts in the atrium and propagates from the atrium toward the ventricle, evident as each atrial calcium wave is followed by a calcium wave through the ventricle. In addition, each calcium wave is accompanied by cardiac chamber contraction (not illustrated), indicating that cardiac excitation in VAPA/VAPB morphants is appropriately initiated in the atrium but with a reduced frequency compared with controls. As described earlier, in 20% of all ^MO^VAPA/B-injected embryos, conduction abnormalities were observed (*n* = 20). We found atrio-ventricular blocks, in which not every atrial excitation causes ventricular excitation, as evident from Fig. 5*F* illustrating a 2:1 atrio-ventricular block. The excitation-contraction coupling of VAPA/VAPB morphants was intact, indicating that knock-down of VAPA/VAPB only impairs the initiation of excitation in the atrium and the atrio-ventricular conduction, which is likely to correlate to a QT time prolongation in mice.

Tail-cuff measurements of VAPB^−/−^ mice detected a prominent heart rate reduction (Fig. 5*G*) that did not, however, result in altered blood pressure (Supplemental Fig. 9). Surface ECG recordings of VAPB^−/−^ mice (Fig. 5*H–N* and Supplemental Figs. 10 and 11) revealed a sino-atrial bradycardia (Fig. 5*H, I*), as heart rates were reduced while the duration of the PQ interval, corresponding to atrio-ventricular conduction, was unchanged (Fig. 5*J, K*). However, the QTc interval (Fig. 5*L*) was prolonged by ∼8%. In addition, the T_peak_-T_end_ (T_p_-T_e_c) interval (Fig. 5*M*) was prolonged and the JT-wave amplitude was increased (Fig. 5*N* and Supplemental Fig. 11), effects that were also observed for mice with genetically impaired HCN channels (38).

### VAPB modulates *I*_f_ of spontaneously beating cardiac HL-1 cells

**Figure 6:**
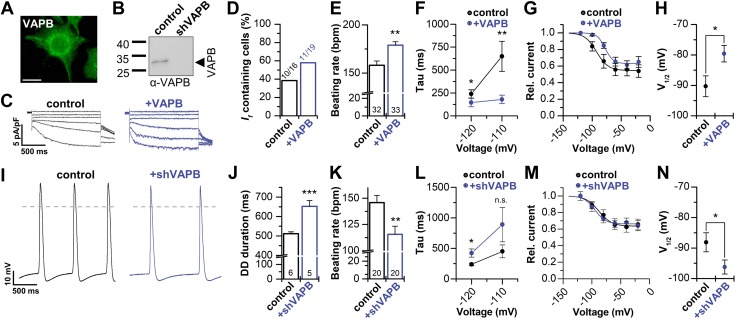
VAPB modulates *I*_f_ of spontaneously beating cardiac HL-1 cells. *A*) Immunocytochemistry of VAPB in HL-1 cells. Scale bar, 20 µm *B*) Western blot illustrating the knock-down of VAPB expression in HL-1 cells by shRNA transfection. Control, HL-1 cells transfected with scrambled shRNA. *C*) Representative *I_f_* currents of HL-1 cells under control conditions and after VAPB transfection. *D*) Percentage of HL-1 cells containing *I*_f_ under control conditions (38%) and after VAPB transfection (58%). *E*) Beating frequency under control conditions (158 ± 4) and after VAPB transfection (179 ± 4), analyzed by optical counting of contractions in original Claycomb medium containing norepinephrine (60). *F*) Accelerated activation kinetics of VAPB-transfected HL-1 cells (*n* = 9–10). *G*) Activation curves of HL-1 cells under control conditions (*n* = 10) and after VAPB transfection (*n* = 11). *H*) Positive shift in the *V*_1/2_ of activation of *I*_f_ recorded in VAPB transfected HL-1 cells. Control (scrambled shRNA), −90.3 ± 3.4 mV (*n* = 10); VAPB transfected, −79.6 ± 2.7 mV (*n* = 11). *I*) Representative action potential measurements of wild-type HL-1 and shRNA transfected cells (shVAPB). *J*) Analysis of the diastolic depolarization (DD duration). *K*) Beating frequency of HL-1 cells under control conditions and after VAPB knock-down. *L*) VAPB knock-down slows the activation kinetics (*n* = 5) of endogenous *I*_f_ currents. *M*) Transfection of shRNA (*n* = 5) shifts the voltage-dependence of activation (*V*_1/2_) of *I*_f_ to more negative potentials (*n* = 6). *N*) *V*_1/2_ values for control (scrambled shRNA) were −88.2 ± 3.1 mV (*n* = 6) and for shRNA-transfection (shVAPB), −96.2 ± 2.3 mV (*n* = 5), respectively. (*I*, *J*), Scrambled shRNA was used as control. All data are presented as means ± sem. The number of experiments (*n*) is indicated in the bar graphs. N.s., not significant. *P < 0.05, **P < 0.01, ***P < 0.001 [unpaired Student’s *t* test (*D*, *G*, *H*, *M*) or Mann-Whitney *U* test (*E*, *F*, *J*–*L*, *N*)

We next studied the effects of VAPB in HL-1 cells, a spontaneously beating sino-atrial node-like cardiomyocyte cell line (39). HL-1 cells endogenously express VAPB (**Fig. 6*A***), which was efficiently diminished by short hairpin RNA (shRNA)-mediated knock-down (Fig. 6*B*). Overexpression of VAPB in HL-1 cells accelerated spontaneous beating rates (Fig. 6*E*), consistent with increased *I*_f_ amplitudes. As previously reported, not all HL-1 cells express macroscopic *I*_f_ currents (28). However, we found *I*_f_-containing cells 1.5-fold more often when HL-1 cells were transfected with VAPB (Fig. 6*D*). Strikingly, the kinetics of channel activation was accelerated by VAPB (Fig. 6*C, F*), and the voltage dependence of *I*_f_ was shifted by 10 mV (Fig. 6*G, H*). This effect can be explained by increased HCN1 and HCN2 current amplitudes and thus a decreased relative contribution of HCN4 to *I*_f_ in this cell line. Conversely, shRNA-mediated knock-down of VAPB significantly reduced the spontaneous action potential frequency (Fig. 6*I, K*) by a prolongation of the diastolic depolarization phase (Fig. 6*I, J*). Norepinephrine treatment of HL-1 cells increased the beating frequency and, also under these conditions, VAPB knock-down strongly reduced the beating frequency (Supplemental Fig. 12). Strikingly, VAPB knock-down had the opposite effects on current kinetics and voltage dependence, as VAPB overexpression. VAPB knock-down slowed the activation time constants (Fig. 6*L*) and led to more hyperpolarized *V*_1/2_ values (−9 mV) (Fig. 6*M, N*). These *in vitro* data confirm that VAPB serves to increase macroscopic *I*_f_ currents in sino-atrial cells, providing an explanation for the sino-atrial bradycardia observed in VAPB^−/−^ mice.

## DISCUSSION

Because VAPB specifically serves to increase both HCN1 and HCN2 current amplitudes, the knock-out of VAPB results in a similar or even more severe sino-atrial bradycardia than knock-out of the murine HCN1 or HCN2, respectively (40, 41). All HCN isoforms are expressed in the working myocardium (38), and an unexpected contribution to the late repolarization phase of the ventricular action potential has been postulated (38, 42, 43). In this case, HCN3^−/−^ mice presented with strong changes in T-wave morphology and a mild (12%) prolongation of the QT interval (38). The transmural gradient, with an increased T-wave amplitude, resulted from a shortening of the action potential duration selective for epicardial cardiomyocytes. This transmural effect on action potential duration was also present in HCN1^−/−^ mice. The mechanism behind this epicardial-specific shortening of the action potential duration, and why concomitantly a QT-interval prolongation is present, remained elusive. Strikingly, VAPB^−/−^ mice presented a similar phenotype, indicating that VAPB is also an essential component of the ventricular *I*_f_ channel complex. Thus, in the future, detailed regional action potential studies are indicated in VAPB^−/−^ mice to further investigate this phenomenon. In addition, VAPB might be relevant for inherited forms of ventricular arrhythmias or for arrhythmias in heart failure, in which increased ventricular *I*_f_ current densities are discussed as a major trigger (43). Thus far, congenital arrhythmias have only been associated with HCN4, the predominant sino-atrial HCN transcript. It is possible that human mutations affecting either HCN1 or HCN2 are compensated by the other isoform, preventing a cardiac phenotype. It is conceivable that only a combined reduction of HCN1 and HCN2 current in sino-atrial cells leads to a cardiac phenotype. Similar to Bartter syndrome type IV, in which the auditory phenotype is only observed when Barttin, the subunit for both ClC-K chloride channels (ClC-Ka and ClC-Kb), is genetically impaired (44), mutations in VAPB might be responsible for cardiac arrhythmias that were not found for HCN1 or HCN2 channels alone.

A major question arising from our results is whether this novel function of VAPB in regulating HCN1 and HCN2 activities could be involved in motor neuron disease. Indeed, gene mutations of *VAPB* have been found in several families developing ALS8, a motor neuron disease also affecting the autonomic nervous system (33, 45). Loss of the VAPB protein occurs in neurons from patients with ALS8 (46), as well as in motor neurons of patients with sporadic ALS (47). Importantly, the P56S mutation of VAPB leads to a loss of HCN activity (Fig. 1*T*). Thus, mutant VAPB, as well as loss of VAPB, might lower dendritic *I*_h_, primarily encoded by HCN1 and HCN2 in motor neurons (48, 49), thereby decreasing excitability of motor neurons. Indeed, increasing motor neuron excitability is protective for motor neurons during ALS (50, 51) (as reviewed by Roselli *et al.* [52]). Thus, the loss of VAPB in motor neurons, although not sufficient to trigger ALS in mice or zebrafish (27), could substantially contribute to motor neuron degeneration through decreased excitability. Conversely, the effects might vary according to neuron type and brain region. For instance, in the cortex and hippocampus, loss of dendritically localized *I*_h_ is expected to lead to a hyperexcitability.

Arrhythmias and sudden cardiac death are common in ALS (53). Sudden cardiac death is responsible for ∼20% of the death cases in patients with ALS (54, 55). Patients with ALS experience prolonged QTc intervals with increased QTc dispersion (54), a dysregulation of the heart rate (56), and have a 25% increased prevalence for atrio-ventricular block (57). However, VAPB mutations are very rare, and only 1 Brazilian family has been investigated in-depth (58). In this family, there was no evidence of cardiac arrhythmia, and, to our knowledge, no patient with a VAPB mutation and typical ALS, as described by Nishimura *et al.* (33), has been studied for cardiac function. Conversely, because VAPA and VAPB are reduced in patients with ALS (35), a dysregulation of HCN channels might also be relevant for sporadic or other familial forms of ALS. The arrhythmias and effects on QTc interval are believed to primarily result from a degeneration of sympathetic neurons of the intermediolateral nucleus (54). Thus, a reduced sympathetic activity could mask an intrinsic bradycardia, due to a dysregulation of HCN channels. In addition, in patients with ALS, afflicted by a severe and life-threatening disorder and with the motor symptoms dominating, a bradycardia with less psychologic strain might have frequently not been brought to the attention of the physicians.

HCN channels in the brain are modulated by Trip8b (PEX5R) (59), which reduces the cAMP responsiveness of HCN2 and HCN4 (12) but not of HCN1 channels (60). In addition, however, Trip8b also produces a larger increase in maximal *I*_h_ by cAMP (61). The proximal N terminus of Trip8b is alternatively spliced and differentially controls HCN trafficking and function, with the most prominent neuronal Trip8b isoform enhancing HCN1 channel surface expression (62). This HCN channel modulation is mediated by an interaction with the CNBD (63) or the distal C terminus (59), whereas the entire C terminus is dispensable for the interaction of HCN1 and HCN2 with VAPB. Thus, although VAPB and Trip8b both serve to increase surface expression of HCN channels, they act *via* different binding domains. In addition, VAPB does not cause a modulation of the cAMP responsiveness of HCN channels. Although the 2 proteins bind to different regions of the pacemaker channels, it prompts analysis of whether these subunits act in a synergistic manner or whether there is functional interplay with the Trip8b effects on channel gating.

We summarize that VAPB is an essential regulator of neuronal and cardiac pacemaker channels, modulating the surface expression and cellular localization of HCN2 and HCN1 channels. Therefore, VAPB is likely to play a role in HCN-mediated processes such as the regulation of cellular excitability, dendritic integration, and transmission of synaptic potentials (64). VAPB modulation of HCN channels might be highly relevant for our understanding of many diseases such as cardiac arrhythmias, epilepsies, inflammatory or neuropathic pain, and ALS (33, 64).

## Supplementary Material

This article includes supplemental data. Please visit *http://www.fasebj.org* to obtain this information.

## Abbreviations

ALS: amyotrophic lateral sclerosis
CC: coiled-coil
CNBD: cyclic nucleotide–binding domain
DIG: digoxigenin
ECG: electrocardiogram
GFP: green fluorescent protein
GST: glutathione S-transferase
HA: hemagglutinin
HCN: hyperpolarization-activated cyclic nucleotide-gated
hpf: hours postfertilization
iPSC-CM: induced pluripotent stem cell–derived cardiomyocyte
ISH: *in situ* hybridization
*I–V*: current–voltage
MO: morpholino-modified antisense oligonucleotide
MSP: major sperm region
Pir: piriform cortex
shRNA: short hairpin RNA
SSC: saline-sodium citrate
SNARE: soluble *N*-ethylmaleimide-sensitive factor attachment protein receptor
TEVC: two-electrode voltage-clamp
TM: transmembrane
VAMP: vesicle-associated membrane protein
VAPB: VAMP-associated protein B
Y2H: yeast 2-hybrid
