# Supplementary material for: The VAMP-associated protein VAPB is required for cardiac and neuronal pacemaker channel function
Source: FASEB J. 2018 Jun 7;32(11):6159–73. doi: 10.1096/fj.201800246R (PMC6629115; doi:10.1096/fj.201800246R)
Supplement: Supplementary file 1 [file fj.201800246R.sd1.pdf]

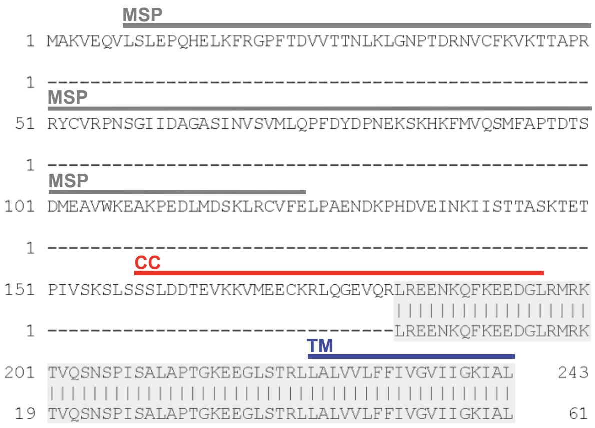

Supplemental Figure 1. VAPB fragment isolated with HCN2 from a human brain cDNA library. Alignment of VAPB and the VAPB fragment identified with the Y2H screen (highlighted in light gray). The VAPB fragment includes parts of the coiled-coil domain (CC, red) and the complete transmembrane domain (TM, blue), but lacks the major sperm domain (MSP, dark gray).

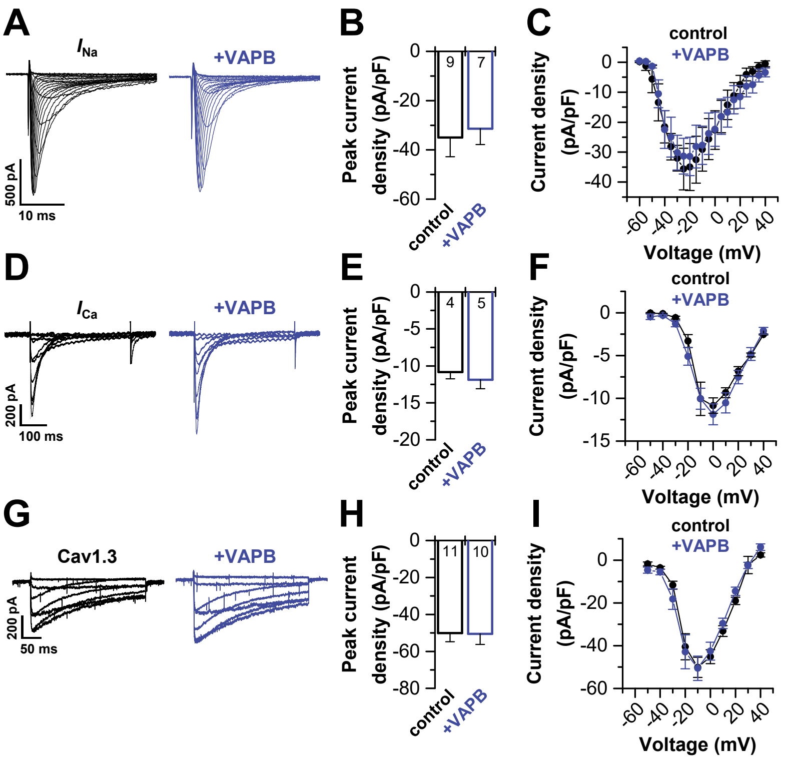

Supplemental Figure 2. VAPB does not alter sodium (I_{Na}) and calcium (I_{Ca}) currents in mouse iPSC cardiomyocytes (iPSC-CM) or currents of Cav1.3 expressed in HEK293 cells. *A*) Representative current traces of endogenous sodium currents (I_{Na}) present in mouse iPSC-CMs under control conditions (transfected with empty vectors) *versus* cells transfected with VAPB. *B*) Analyses of the peak current densities showed no significant differences between control iPSC-CMs and iPSC-CMs expressing VAPB. Peak current densities were: control -32.1 ± 4.3 pA/pF and +VAPB -30.1 ± 5.8 pA/pF , respectively. *C*) Analyses of the current densities of I_{Na} in iPSC-CMs alone (n = 9) or after transfection of VAPB (n = 7) for different voltages (*IV*s). *D*) Representative current traces of endogenous calcium currents (I_{Ca}) present in mouse iPSC-CMs under control conditions (transfected with empty vectors) *versus* cells transfected with VAPB. *E*) Analyses of the peak current densities showed no significant differences between control iPSC-CMs and iPSC-CMs expressing VAPB. Peak current densities were: control -10.9 ± 0.9 pA/pF and +VAPB -11.9 ± 1.2 pA/pF, respectively. *F*) Analyses of the current densities of I_{Ca} in iPSC-CMs alone (n = 4) or after transfection of VAPB (n = 5) for different voltages (*IV*s). *G*) Representative current traces of HEK293 cells transfected with Cav1.3 α_1-, β_{2b}-, $\alpha_2\delta_1$-subunits, pEGFP and either empty pcDNA3.1(+) (control) or VAPB pcDNA3.1(+). *H*) Analyses of the peak current densities: control -50.1 ± 4.7 pA/pF and +VAPB -50.5 ± 5.7 pA/pF. *I*) Analyses of the current densities of Cav1.3 expressed alone (n = 11) or in the presence of VAPB (n = 10) for different voltages (*IV*s). All data are presented as mean ± s.e.m.. The number of experiments (n) are indicated in the bar graphs. For more information see also Supplementary Methods.

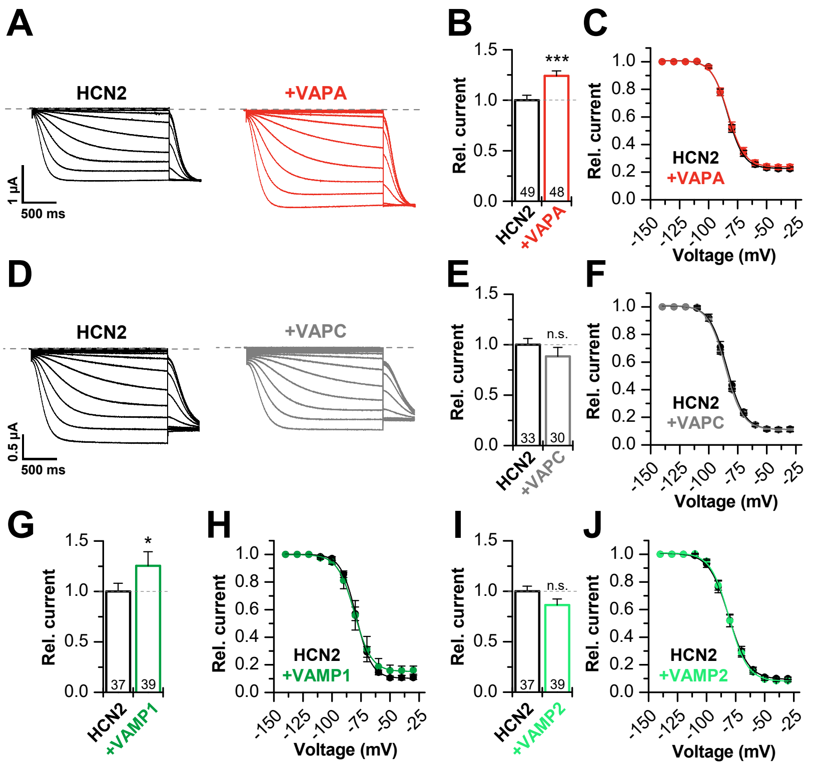

Supplemental Figure 3. VAPA, VAPC, VAMP1 and VAMP2 display differential effects on HCN2 current amplitudes. *A*) Representative currents of oocytes injected with HCN2 alone or co-injected with VAPA. *B*) Analysis of the HCN2 current amplitudes after co-expression with VAPA (1.24 ± 0.05, red), analyzed at -130 mV. *C*) $V_{1/2}$ values of HCN2 expressed alone (*n* = 13) or co-expressed with VAPA (*n* = 12). *D*) Representative currents of oocytes injected with HCN2 alone or co-injected with VAPC. *E*) Co-expression of HCN2 with VAPC did not alter HCN2 current amplitudes (0.88 ± 0.09, gray), analyzed at -130 mV. *F*) $V_{1/2}$ values of HCN2 alone (*n* = 33) and after co-expression with VAPC (*n* = 30). *G*) and *H*), Co-expression of HCN2 with VAMP1 increased current amplitudes (1.26 ± 0.14), analyzed at -130 mV, with no effect on voltage-dependence of activation ($V_{1/2}$) (HCN2: *n* = 5, HCN2+VAMP1 *n* = 3). *I*) and *J*), Co-expression with VAMP2 did not alter HCN2 current amplitudes, analyzed at -130 mV or voltage-dependence (HCN2: *n* = 24, HCN2+VAMP2 *n* = 23). All data are presented as mean ± s.e.m.. The number of experiments (*n*) are indicated in the bar graphs. *, P < 0.05 and ***, P < 0.001 using an unpaired Student´s T-test, except for panels *B*), *G*) and *I*), using a Mann-Whitney-U-test.

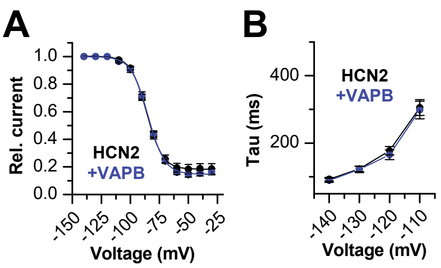

Supplemental Figure 4. VAPB does not alter the voltage-dependence or activation kinetics of HCN2. *A*) Voltage dependence of HCN2 (*n* = 20) and HCN2 co-expressed with VAPB (*n* = 30) and *B*) the analysis of the activation kinetics at three different membrane potentials. All data are presented as mean ± s.e.m..

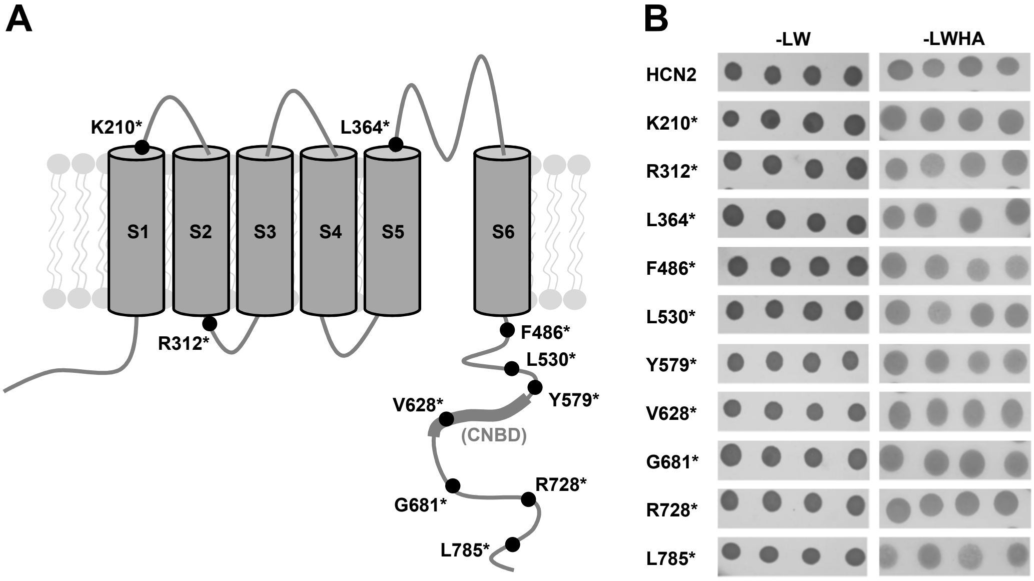

Supplemental Figure 5. Y2H direct interaction assay revealed that the C-terminus of HCN2 is dispensable for the interaction with VAPB. *A*) Localization of all HCN2 truncation mutants studied in the Y2H direct interaction assay. *B*) Growth on -LW drop out medium indicates an uptake of both constructs into the yeast, as transformation control for VAPB and HCN2. All mutants were capable to interact with VAPB, as the yeast clones were growing on minimal media lacking -LWHA. For all combinations the yeast was spotted in four replicates.

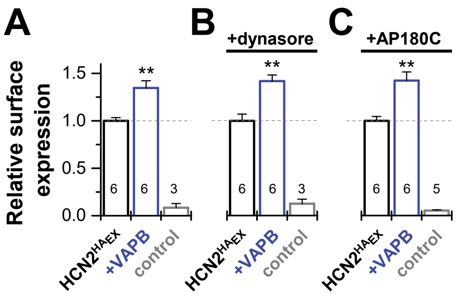

Supplemental Figure 6. VAPB increases the forward transport of HCN2 channels to the surface membrane. *A*) Relative changes in surface expression of extracellularly HA-tagged HCN2 ($HCN2^{HA_{Ex}}$), transfected in HeLa cells (1 µg), by the co-transfection of VAPB (1 µg). Values are normalized to the relative light units of $HCN2^{HA_{Ex}}$. *B*) Same experiments as in *A*), analyzing the relative change in surface expression, but all cells were incubated in 40 µM dynasore after transfection. *C*) Same experiments as in *A*), analyzing the relative change in surface expression, but all cells were co-transfected with AP180C (0.25 µg). Chemiluminescence assays were performed 24 h after transfection. All data are presented as mean ± s.e.m.. Experiments were performed from three independent transfections and the number of separate dishes are indicated (*n*) in the bar graphs. **, P < 0.01; ***, P < 0.001 using an unpaired Student´s T-test.

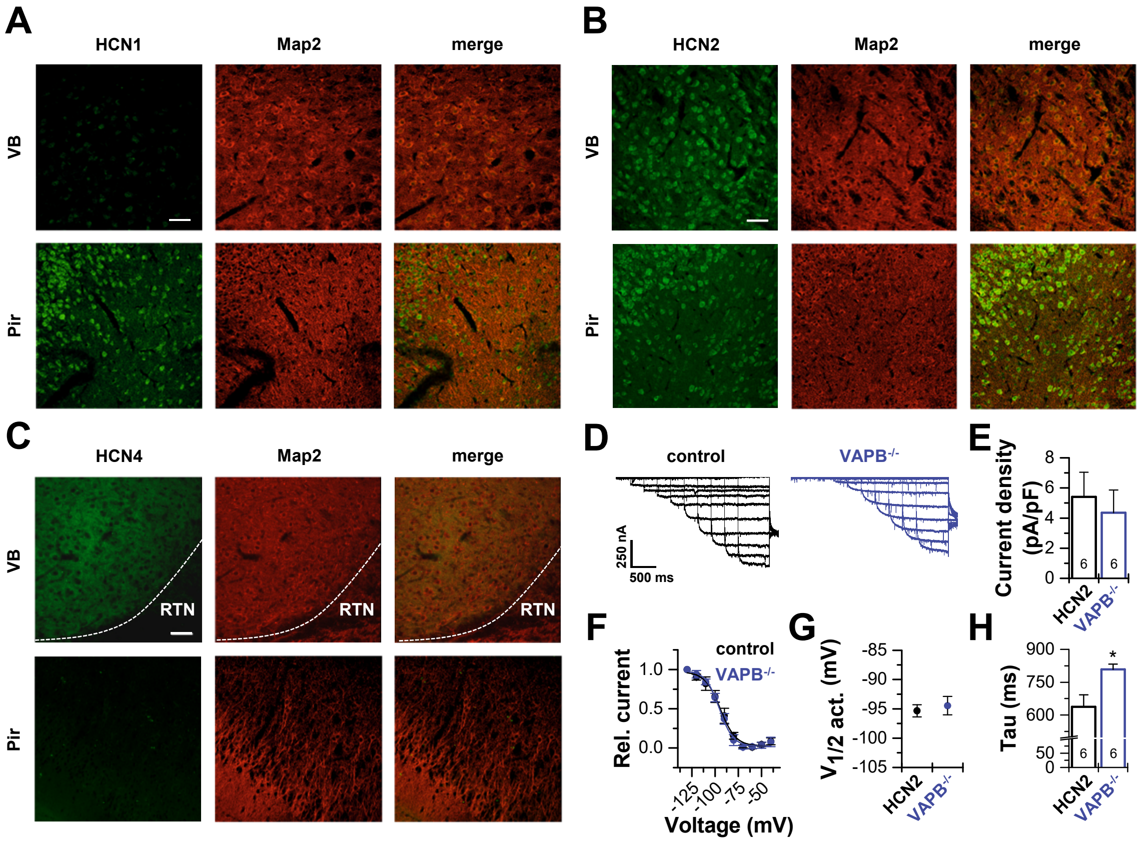

Supplemental Figure 7. Specific expression pattern of HCN isoforms and the role of VAPB for the I_h current in the piriform cortex. *A*) Brain slices were stained with primary antibodies directed against neuronal marker Map2 (guinea pig-anti-Map2, 1:100, depicted in red) and HCN1 subunit (1:200, depicted in green) in piriform cortex (Pir) of control (C57BL/6NTac) mice. The ventrobasal complex of the thalamus (VB) is shown for comparison. Note the lower expression of HCN1 in VB (upper panel, left) compared to Pir cortex (lower panel, left). *B*) Specific labeling of HCN2 (depicted in green, 1,200) and Map2 (1:100, depicted in red) in VB (upper panel) and Pir cortex (lower panel). *C*) Specific labeling of HCN4 (1:200) and Map2 (1:100) in VB (upper panel) and Pir cortex (lower panel). Dashed lines indicate the location of the reticular thalamic nucleus (RTN). Compared to the VB (upper panel, in green), Pir cortex (lower panel, in green) shows a lower expression level for HCN4 subunit. *D*) Representative traces of I_h recorded under voltage-clamp conditions from pyramidal neurons of the Pir cortex in control (+/+) and VAPB$^{-/-}$ mice. The last step illustrated is to -130 mV. *E*) Bar graph comparing the I_h current density in these neurons. *F*) Mean steady-state I_h activation curves in pyramidal neurons of Pir cortex in control (in black, n = 6 cells) and VAPB$^{-/-}$ mice (in blue, n = 6 cells). *G*) Graph comparing the half maximal activation of I_h ($V_{1/2}$) in control ($V_{1/2}$ = -95.4 ± 1.0 mV, n = 6 cells) and VAPB$^{-/-}$ ($V_{1/2}$ = -94.5 ± 1.6 mV, n = 6 cells) pyramidal cells. *H*) Bar graphs indicating the increase in slow activation time-constant of I_h in VAPB$^{-/-}$ pyramidal neurons compared to control cells. All data are presented as mean ± s.e.m.. The number of experiments (n) are indicated in the bar graphs. *, $P < 0.05$ using an unpaired Student´s T-test. Scale bars for *A*)-*C*), 50 µm. For more information see also Supplemental Methods.

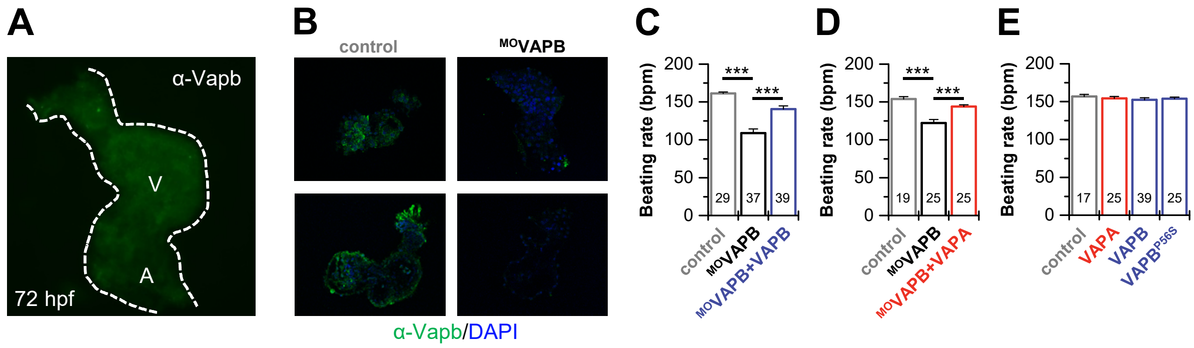

Supplemental Figure 8. Cardiac expression of VAPB in embryonic zebrafish hearts, rescue experiments after MO-VAPB knock-down and overexpression of VAPB^{P56S}. *A*) Immunostaining with an α-VAPB antibody (see Supplementary Methods) showing an uniform expression of VAPB in embryonic zebrafish hearts at 72 hours post fertilization. A, atrium. V, ventricle. *B*) Two representative embryonic zebrafish hearts stained against VAPB, either after injection with control morpholinos or with the VAPB knock-down morpholino, respectively. The embryonic zebrafish heart was co-stained against DAPI to show the cell nuclei. The experiments illustrate an efficient knock-down of VAPB and specificity of the antibody. *C*) After morpholino-antisense mediated VAPB knock-down (^{MO}VAPB), the injection of cRNA encoding for either VAPB or *D*) VAPA rescued the reduction in embryonic heart rates. Data were obtained from four and three independent batches of injections, respectively. *E*) The injection of cRNA for the human VAPA or VAPB did not alter embryonic heart rates. Moreover, injection of VAPB^{P56S} cRNA is likely to not act in a dominant-negative manner on zebrafish I_f channels, as the heart rates are not altered. Data were obtained from three independent batches of injections. All data are presented as mean ± s.e.m.. The number of experiments (*n*) are indicated in the bar graphs. ***, P < 0.001 using an unpaired Welch´s T-test.

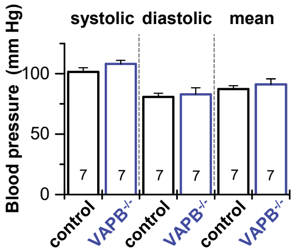

Supplemental Figure 9. Normal blood pressure in VAPB$^{-/-}$ mice. Tail-cuff measurements indicate that there are no changes in systolic and diastolic blood pressure in VAPB$^{-/-}$ mice. All data are presented as mean ± s.e.m.. The number of animals (*n*) are indicated in the respective bar graphs.

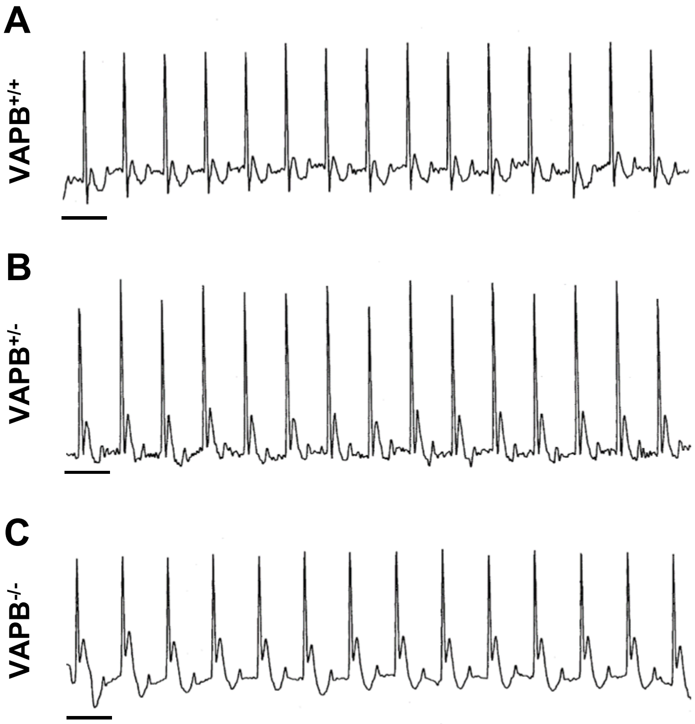

Supplemental Figure 10. Surface electrocardiograms (ECG) (Lead I) of wild-type, VAPB$^{+/-}$ and VAPB$^{-/-}$ mice. Representative ECGs of adult unsedated mice, using an EMKA ECG tunnel system. *A*) Wild-type mice, *B*) heterozygous (VAPB$^{+/-}$) and *C*), homozygous (VAPB$^{-/-}$) knock-out animals. Scale bars, 100 ms.

A

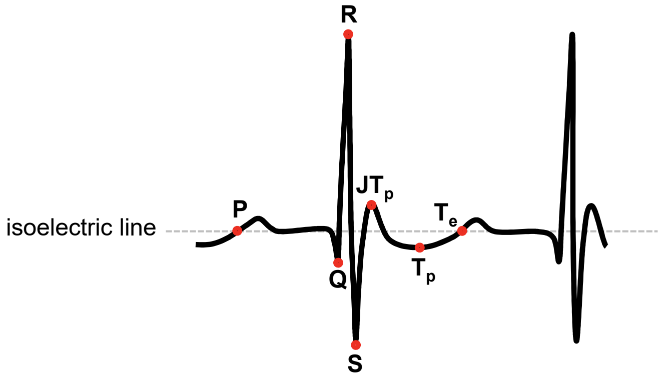

B

Genotype	*n*	Sex (m/f)	Age (weeks)	HR (bpm)	PQ (ms)	QRS (ms)	QT (ms)	QTc (ms)	S-T$_e$ (ms)	S-T$_e$c (ms)	JT$_p$ ampl. (%)	JT$_p$-T$_e$ (ms)	JT$_p$-T$_e$c (ms)	T$_p$-T$_e$ (ms)	T$_p$-T$_e$c (ms)
+/+	8	6/2	67.1 ± 11.7	734.8 ± 4.7	37.7 ± 1.1	11.6 ± 0.4	44.0 ± 1.4	45.1 ± 1.3	33.3 ± 1.1	34.1 ± 1.2	13.0 ± 2.5	26.3 ± 1.4	26.9 ± 1.5	9.4 ± 1.8	9.6 ± 1.8
+/-	10	8/2	63.9 ± 11.3	729.0 ± 10.8	37.7 ± 1.6	11.1 ± 0.4	44.7 ± 1.28	45.6 ± 1.2	34.0 ± 1.4	34.6 ± 1.4	18.1 ± 5.0	27.1 ± 1.2	27.6 ± 1.1	7.9 ± 1.1	8.0 ± 1.2
-/-	13	9/4	49.2 ± 13.0	686.4 ± 13.9	36.4 ± 1.5	11.5 ± 0.4	49.3 ± 1.1	48.7 ± 0.7	38.8 ± 1.2	38.4 ± 1.0	21.5 ± 4.2	31.4 ± 1.2	31.0 ± 1.0	13.8 ± 1.0	13.6 ± 1.0

Blue boxes: *$p < 0.05$ vs. VAPB$^{+/+}$ and VAPB$^{+/-}$*, ***Red box:*** *$p < 0.05$ vs. VAPB$^{+/+}$*

Supplementary Figure 11. ECG parameters of wild-type (+/+), heterozygous (+/-) and homozygous (-/-) VAPB knock-out mice. *A*) Schematic representation of the ECG. (P) the beginning of the P wave, (JT$_p$) JT peak, first max deflection after the QRS complex (usually positive in lead I), (T$_p$) T-wave peak, greatest late T wave amplitude (usually negative in lead I), (T$_e$) T wave end, return of the last T wave deviation to the isoelectric line. *B*) Table of the ECG parameters (HR) heart rate, (PQ) beginning of P wave to Q, (QRS) peak of Q to S, (QT) Q to end of T wave, (QTc) rate corrected QT intervall ($QT/\sqrt{700/HR}$, same for other rate corrections), (S-T$_e$) S to end of T wave, (JT$_p$ ampl.) value of relative JT$_p$ amplitude normalized to the respective R peak in percent, (JT$_p$-T$_e$) JT$_p$ to end of T wave, (T$_p$-T$_e$) T$_p$ to end of T wave. All data are presented as mean ± s.e.m.. Significance was probed using an unpaired Student´s T-test or or Welch´s T-test.

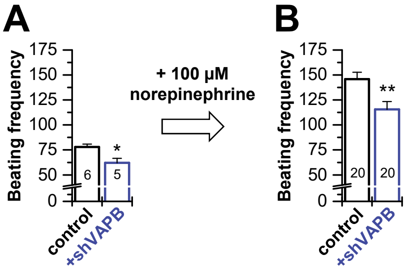

Supplemental Figure 12. Knock-down of VAPB reduces beating frequency under basal conditions and after isoproterenol stimulation. *A*) Patch clamp recordings reveal a reduced action potential (AP) frequency of HL-1 cells transfected with shRNA against VAPB. *B*) Treatment of cultured HL-1 cells with 100 µM norepinephrine-containing Claycomb medium increased spontaneous beating frequency, analyzed by optical counting of contractions under video analyses. Also under stimulated conditions shRNA-mediated knock-down reduced spontaneous beating frequency. All data are presented as S.E.M.. The number of experiments *(n)* are indicated in the respective bar graphs. *, $P < 0.05$; **, $P < 0.01$ using an unpaired Student´s T-test, except for panel *B*) using a Mann-Whitney-U-test.

Supplemental Table 1. Primers used for *in situ* hybridization of HCN2, VAPA and VAPB.

Name	Primer	Amplicon length (basepairs)
**HCN2**	Forward: GAGCAATACATGTCCTTCCACA<br>Reverse: GTTCTTCTTGCCTATGCGATCT	522
**VAPA**	Forward: GGATCCACCTCAGCCGTGTCCT<br>Reverse: AGAGGCACCATTTCCCCCACAGA	591
**VAPB**	Forward: GCCACCGTGCTCACAGATGGAC<br>Reverse: ATCGCCCAGCCTCACCTATGCT	1043
